# Destruction and regrowth of lithospheric mantle beneath large igneous provinces

**DOI:** 10.1126/sciadv.adf6216

**Published:** 2023-09-06

**Authors:** Simon N. Stephenson, Patrick W. Ball, Fred D. Richards

**Affiliations:** ^1^ Department of Earth Sciences, University of Oxford, Oxford, UK.; ^2^ Department of Geosciences, Colorado State University, Fort Collins, CO, USA.; ^3^ Department of Earth Science and Engineering, Imperial College London, London, UK.

## Abstract

Large igneous provinces (LIPs) are formed by enormous (i.e., frequently >10^6^ km^3^) but short-lived magmatic events that have profound effects upon global geodynamic, tectonic, and environmental processes. Lithospheric structure is known to modulate mantle melting, yet its evolution during and after such dramatic periods of magmatism is poorly constrained. Using geochemical and seismological observations, we find that magmatism is associated with thin (i.e., ≲80 km) lithosphere and we reveal a striking positive correlation between the thickness of modern-day lithosphere beneath LIPs and time since eruption. Oceanic lithosphere rethickens to 125 km, while continental regions reach >190 km. Our results point to systematic destruction and subsequent regrowth of lithospheric mantle during and after LIP emplacement and recratonization of the continents following eruption. These insights have implications for the stability, age, and composition of ancient, thick, and chemically distinct lithospheric roots, the distribution of economic resources, and emissions of chemical species that force catastrophic environmental change.

## Teaser

Lithospheric mantle is destroyed during magmatism, then regrows after magmatism ceases, reaching cratonic thickness on continents.

## INTRODUCTION

The thermomechanical structure of the lithospheric plate and its interaction with the underlying asthenosphere influence the evolution of Earth’s topography, plate motions and deformation, the pattern of mantle convection, and the location and style of volcanism (*1*–*6*). Consequently, lithospheric evolution drives erosional and depositional processes, controls the distribution of economic resources, and influences climatic and environmental conditions through space and time (*7*–*9*). Geological and geophysical observations provide constraints on present-day lithospheric structure, but only snapshots of its variations through time [e.g., (*8*, *10*–*14*)].

It is often assumed that the lithospheric structure of ancient continental interiors (i.e., cratons) is stable and unchanging over very long time periods, yet there is a paucity of data with which to test this hypothesis [see (*15*) for a comprehensive review]. This interpretation is generally based on the exposure of exclusively ancient, undeformed shields at low elevations with low relief (*16*). These shields overlie a thick, depleted lithospheric mantle (i.e., up to ∼250 km in thickness) that is inherently buoyant, viscous, and difficult to convectively remove (*4*, *16*, *17*). However, several of these cratons are capped by Phanerozoic large igneous provinces (LIPs)—rare, but giant outpourings of lava (>10^6^ km^3^) that were extruded within geologically short time frames (<5 Ma)—which are commonly linked to the arrival of plume heads beneath the plate (*18*). For example, the Siberian Traps, Central Atlantic Magmatic Province, and Karoo flood basalts overlie the Siberian, West African, and Kalahari cratons, respectively. This association calls into question the assumption that cratonic lithosphere is always thick and stable since a thin tectonic lid is required to generate large volumes of melt, and present-day magmatism is strongly associated with lithosphere <100 km thick (*1*, *6*, *19*–*21*).

In this contribution, we attempt to reconcile these seemingly contradictory observations by tracking lithospheric evolution during and after magmatism. We begin by comparing the locations and ages of modern and ancient oceanic islands, seamounts, and plateaux to estimates of plate age and lithospheric thickness. Subsequently, this analysis is extended into continental regions, where we develop conceptual and numerical models to explain the lithospheric response to intraplate magmatic events. Finally, we describe the implications of our results for the thermochemical structure of cratonic lithosphere and their use in addressing several key geodynamic, economic, and environmental questions.

## RESULTS

### Oceanic intraplate magmatism

#### Recent intraplate magmatism

Our starting point is to explore lithospheric thickness beneath modern-day intraplate magmatic provinces. In the oceanic realm, the lithospheric mantle cools and thickens away from mid-ocean ridges in a predictable way (*10*, *22*, *23*). This phenomenon is one of the most widely accepted and well studied in global geophysics. Usefully, it allows expected time-dependent deepening of the lithosphere-asthenosphere boundary (LAB) to be used as a reference template against which deviations in lithospheric thickness can be defined and explored.

**Fig. 1.**
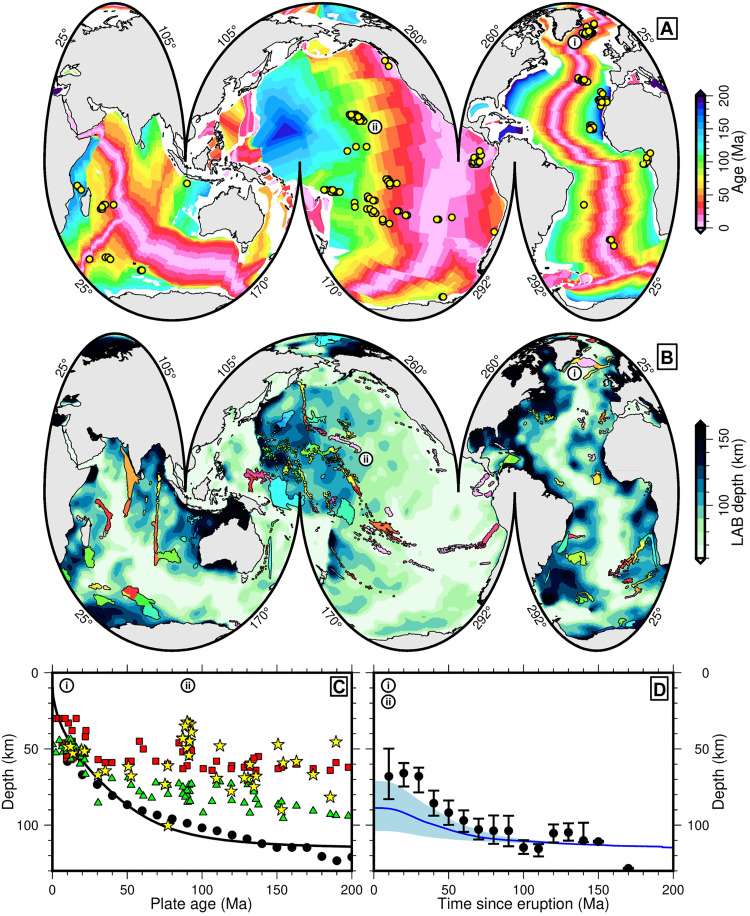
Distribution of oceanic magmatic provinces. (**A**) Oceanic crustal age (*27*, *85*). Yellow circles: locations of intraplate magmatic provinces (*20*). (i) and (ii): locations of Icelandic and Hawaiian hot spots. (**B**) Oceanic LAB depth (*8*). Colored polygons: locations of oceanic magmatic provinces (i.e., ocean islands, seamounts, and plateaux) colored by age. (**C**) Constraints on modern-day LAB depth beneath intraplate magmatic provinces as a function of underlying oceanic crustal age. Red squares: LAB depth determined by inverse modeling of REE compositions of mafic rocks (*20*); green triangles: LAB depth determined by tomographically-derived LAB depth model shown in (B) (*8*, *75*); yellow stars: LAB depth estimated by finding magmatic equilibration pressure and temperature that corresponds to base of lithosphere (this study, see main text and Materials and Methods); (i) and (ii): age of crust underlying Icelandic and Hawaiian hot spots. (**D**) Black circles with error bars: modern LAB depth beneath magmatic provinces as a function of province age; moving median at 10-Ma intervals with a ±10 Ma window; error bars: interquartile range; blue line: median expected distribution of LAB depth beneath LIPs if they were distributed randomly in time and space across oceanic lithosphere assuming plate model of Richards *et al.* (*28*) and plate age model of Müller *et al.* (*27*); blue envelope: interquartile range; (i) and (ii): age of Icelandic and Hawaiian hot spots.

We wish to determine whether intraplate magmatism is associated with deviations from the plate-cooling relationship. To carry out this test, we exploit a global database of intraplate magmatic analyses (*20*). This compilation includes major and trace geochemical compositions of mafic rocks at all locations where intraplate magmatism has occurred within the last 10 Ma and has not since moved further than 400 km from the site of eruption (Fig. 1A). We estimate modern-day depth to the LAB, $z_{\mathrm{LAB}}^{1}$, beneath each recent intraplate magmatic province in three ways. First, $z_{\mathrm{LAB}}^{1}$ beneath each location is extracted from the lithospheric thickness model of Hoggard *et al.* (*8*), which is located by contouring the 1175°C isothermal surface after converting the SL2013sv shear-wave tomographic model into temperature (Fig. 1B). This temperature conversion scheme exploits a calibrated elastic-anelastic parameterization (*24*). Second, we use the lithospheric thickness estimates calculated by inverse modeling of rare earth element (REE) concentrations within mafic intraplate magmas carried out by Ball *et al.* (*20*) (see Materials and Methods). This method is predicated upon the partitioning of REEs between mantle melt and residue during partial melting, where the upper limit of melting is taken to represent the LAB. Finally, we estimate $z_{\mathrm{LAB}}^{1}$ by calculating melt equilibration pressure, *P*_eq_, and temperature, *T*_eq_, of intraplate melts using the thermobarometric scheme developed by Plank and Forsyth (*25*) (dataset S1). This scheme exploits the sensitivity of major oxide phase concentrations within mafic melts to these two properties within the mantle [e.g., (*25*, *26*); see Materials and Methods]. The global database of Ball *et al.* (*20*) is filtered for samples where 9 < MgO < 14.5 wt % to mitigate removal or addition of material by fractionation or contamination. Then, *T*_eq_ and *P*_eq_ are calculated for the remaining samples. We locate samples that equilibrated beneath the LAB by selecting samples where *T*_eq_ > 1175°C. This screening procedure ensures consistency with tomographically determined LAB depths. Next, samples are geographically divided into 1° × 1° bins. We exclude bins with <10 samples since it is unlikely that a very small sample size can be used to accurately locate the base of the lithosphere. Finally, the minimum value of *P*_eq_ is calculated within each bin, which enables an approximation of maximum depth to the LAB (i.e., *z*_LAB_ ≈ 31.4 $P_{\mathrm{eq}}^{\min}$; values of *P*_eq_ and *T*_eq_ are given for each sample in the Supplementary Materials).

Figure 1C and fig. S1 show the three independent estimates of observed $z_{\mathrm{LAB}}^{1}$ beneath recent oceanic intraplate magmatic provinces (dataset S2). We present our results as a function of the age of oceanic crust, *t*_crust_, atop which the magmatic provinces rest (*8*, *27*). All else being equal, $z_{\mathrm{LAB}}^{1}$ beneath these recently erupted provinces should be governed entirely by plate cooling as a function of *t*_crust_. These observed LAB depth constraints are therefore compared to two estimates of expected, unperturbed LAB depth. First, we calculate expected LAB depth using a plate-cooling model constructed by Richards *et al.* (*28*) (i.e., black line in Fig. 1C). Second, we compare to the global median LAB depth as a function of oceanic crustal age determined by Hoggard *et al.* (*8*) (i.e., black circles in Fig. 1C). These two estimates of expected $z_{\mathrm{LAB}}^{1}$ values are in close agreement, although it is important to note that they are not entirely independent of one another [see (*8*, *23*, *24*)].

For magmatic provinces located above young oceanic crust (i.e., *t*_crust_ < 30 Ma old), predictions and observations are in close agreement, indicating that $z_{\mathrm{LAB}}^{1}$ increases from ∼20 to 50 km to 60 to 80 km during this time period. For crustal ages *t*_crust_ > 30 Ma, however, expected and observed LAB depths beneath intraplate magmatic provinces dramatically diverge. For an oceanic crustal age, *t*_crust_ ≳ 30 Ma old, the plate is systematically thinner by 30 to 60 km than would be expected given the crustal age (compare black line and circles with colored symbols in Fig. 1C). These observations suggest that magmatism occurs exclusively where *z*_LAB_ < 100 km, and in most cases only where *z*_LAB_ ≲ 80 km.

To make sense of this relationship, consider first the Icelandic hot spot [(i) in Fig. 1]. This magmatic province straddles the Mid-Atlantic Ridge, where lithospheric mantle is thin or absent due to active seafloor spreading. Consequently, all lithospheric thickness proxies suggest that melts are able to ascend to a shallow level, unimpeded by the presence of lithospheric mantle, before encountering the Moho at depths of ∼15 to 45 km (*29*, *30*). Next, consider the Hawaiian hot spot, which has *t*_crust_ ∼ 90 Ma old [(ii) in Fig. 1]. The LAB ought to be found at $z_{\mathrm{LAB}}^{1}$ ≈ 100 km according to both predicted plate cooling and the global average for oceanic lithosphere of that age. However, major oxide concentrations suggest that Hawaiian melts equilibrate within the asthenosphere to depths as shallow as 30 to 60 km (see yellow stars in Fig. 1C). Furthermore, REE inverse modeling suggests that the top of the asthenospheric melt column is located at 40 to 60 km depth (see red squares in Fig. 1C). Finally, local and global seismic imaging indicates that beneath Hawaii $z_{\mathrm{LAB}}^{1}$ ∼ 40 to 90 km [e.g., (*8*, *31*, *32*); see green triangles in Fig. 1C]. This thinner lithosphere is consistent with flexural and gravimetric observations, as well as with xenolith-based thermobarometric results, which indicate that temperatures of 1000° to 1100°C occur at depths of 45 to 55 km beneath Hawaii (*33*, *34*). In contrast to the Icelandic example, this discrepancy suggests that, locally, $z_{\mathrm{LAB}}^{1}$ is notably shallower than expected beneath Hawaii. Together, these considerations strongly suggest that when the plate thickens beyond 70 to 80 km (i.e., when *t*_crust_ ≳ 30 Ma old), magmatism is associated with marked lithospheric thinning to depths of 60 to 80 km. In other words, although a precise mechanism cannot be discerned, magmatism is associated with the resetting of *z*_LAB_ to depths typically found beneath oceanic crust with an age of ∼20 to 40 Ma.

#### Ancient intraplate magmatism

Following cessation of magmatism, lithosphere that is <80 km thick should rethicken as a result of conductive cooling. Modern-day LAB depth, $z_{\mathrm{LAB}}^{1}$, should therefore be thicker beneath older intraplate magmatic provinces than those erupting at present. We test this hypothesis by substantially updating a global map of oceanic plateaux, islands, and seamounts [(*35*, *36*); Fig. 1B, section S2, and dataset S3]. This database contains polygons outlining these oceanic landforms and an associated eruption age (i.e. time elapsed since eruption), *t*_0_, of the magmatic rocks that form them. Polygons are added and excluded, and their age constraints are updated according to recent literature (see section S3 and fig. S2). We sample $z_{\mathrm{LAB}}^{1}$ at 0.1° × 0.1° intervals beneath each polygon in our updated database. Once again, we exploit estimates of lithospheric thickness calculated by Hoggard *et al.* (*8*). Next, we filter our database to ensure that only the final volcanic episode in a given location is included (dataset S4). Thus, we capture the passive response of the lithosphere to magmatism and avoid contamination by subsequent magmatic events. We remove any location that has experienced another phase of magmatism within a distance of <500 km after >30 Ma of the initial eruption. Note, however, that we do not control for any subsequent amagmatic tectonic thinning or thickening of the lithosphere. We calculate the moving median and interquartile range of $z_{\mathrm{LAB}}^{1}$ values beneath the remaining intraplate magmatic provinces as a function of *t*_0_. In calculating each median value, we weight the $z_{\mathrm{LAB}}^{1}$ value of each point as a function of latitude to avoid biasing moving average estimates toward polar provinces (see Materials and Methods).

On Fig. 1D, the black circles with error bars show observed values of $z_{\mathrm{LAB}}^{1}$ as a function of *t*_0_. The median LAB depth beneath the youngest provinces (i.e., where eruption age, *t*_0_ < 10 Ma) is consistent with the median thickness calculated for the database of Ball *et al.* (*20*), as well as our new thermobarometric constraints (see Materials and Methods; section S2.1.1). We find that there is a positive relationship between province age and LAB depth beneath the province up to an age of ∼80 to 100 Ma, at which point the deepening of $z_{\mathrm{LAB}}^{1}$ flattens off (see black circles with error bars in Fig. 1D). The shape of this relationship resembles a plate cooling model that flattens at a depth consistent with several well-known, published schemes [i.e., 100 to 130 km; Fig. 1 (C and D); e.g., (*10*, *22*, *23*)].

Since older oceanic plate is associated with thicker lithosphere, it is possible that any positive relationship between $z_{\mathrm{LAB}}^{1}$ and *t*_0_ is generated by chance. This general trend could be produced by distributing intraplate magmatism randomly upon a lithospheric plate whose thickness is governed solely by plate cooling as a function of oceanic crustal age. This relationship would arise because provinces with an eruption age of, say, *t*_0_ = 180 Ma can only possibly be found today on old lithosphere, which in the absence of any thermal perturbation would be around 125 km thick [e.g., (*23*)]. However, recent intraplate magmatism (i.e., *t*_0_ = 0 Ma) could theoretically be distributed over lithosphere of thickness ranging from zero at a mid-ocean ridge to 125 km on the abyssal plain. The median LAB depth of this distribution would therefore be shallower than for those locations overlying only older oceanic crust. As a result, even if $z_{\mathrm{LAB}}^{1}$ is controlled only by oceanic crustal age, younger intraplate magmatism is expected to overlie, on average, thinner lithosphere than sites of older magmatism. A sample of young seamounts, islands, and plateaux would also have a broader distribution of $z_{\mathrm{LAB}}^{1}$ values than older provinces. However, if magmatic emplacement is associated with systematic thinning of the plate (i.e., shallowing of the LAB), then observed values of $z_{\mathrm{LAB}}^{1}$ beneath oceanic plateaux, islands, and seamounts ought to be systematically thinner for any *t*_0_ < 80 to 100 Ma than the $z_{\mathrm{LAB}}^{1}$ values expected from plate cooling alone.

To investigate whether temporal trends in $z_{\mathrm{LAB}}^{1}$ provide evidence for magmatism-related lithospheric thinning, we calculate the expected, thermally unperturbed $z_{\mathrm{LAB}}^{1}$ distribution beneath a random sample of oceanic islands, seamounts, and plateaux as a function of *t*_0_ given plate cooling alone (see Materials and Methods). As expected, we find that there is a slight positive relationship between plate age and unperturbed lithospheric thickness beneath ancient oceanic magmatic provinces (see blue line and envelope in Fig. 1D). This predicted distribution is then compared to the observed distribution (i.e., black circles with error bars in Fig. 1D). Up to an eruption age, *t*_0_ ≈ 90 Ma before present, ancient magmatic provinces systematically overlie lithosphere that is ∼20 to 40 km thinner than would be expected if the plate were not perturbed since seafloor spreading (compare black circles and blue line in Fig. 1D). This result is not materially affected by using alternative lithospheric thickness models (see section S4). This finding further supports our earlier insight that intraplate magmatism resets the plate cooling process by thinning the lithospheric mantle. It is also consistent with seminal work by Detrick and Crough (*37*), who showed that ocean island subsidence cannot be explained only by seafloor age. Instead, anomalous subsidence requires lithospheric thinning followed by time-dependent rethickening and/or translation away from a subplate thermal anomaly (*38*). Our results suggest that the thickness of the plate beneath ancient seamounts and plateaux may be better predicted using the eruption age of the province than by oceanic crustal age.

### Continental LIPs

The initial rejuvenation and subsequent progressive thickening of the oceanic lithosphere over time following intraplate volcanism raises an important question: Does continental lithosphere behave in a similar way? Answering this question is complicated by the fact that lithospheric thickness in the continental realm—unlike in the oceans—does not generally increase as a function of crustal age. Nevertheless, Ball *et al.* (*20*) showed that, by extracting LAB depth from the model of Hoggard *et al.* (*8*) and modeling REE concentrations in intraplate magmas, $z_{\mathrm{LAB}}^{1}$ ≲ 100 km beneath modern continental intraplate magmatic provinces. Similarly, our proxy for $z_{\mathrm{LAB}}^{1}$ based on equilibration pressures and temperatures of major oxide phases in mafic melts agrees that almost all modern magmatic provinces are associated with $z_{\mathrm{LAB}}^{1}$ ≲ 80 km [(*20*); see Materials and Methods; section S2 and fig. S1]. Furthermore, White and McKenzie (*39*) modeled REE concentrations in ancient magmatic provinces. They showed that, irrespective of the often substantial thickness of lithosphere beneath each province today, these ancient mafic magmatic rocks were also associated with thin lithosphere at the time of their formation. The question therefore remains as to how the lithosphere evolves following eruption of intraplate magmatic rocks in the continental realm. We now return to our updated database of ancient intraplate magmatic provinces to explore whether the relationship between *z*_LAB_ and intraplate magmatism also applies to the continents.

On the continents, this database is mostly composed of LIPs, a dominance that is particularly acute before Cenozoic times. In the same way as our analysis in the oceanic realm, we have expanded and improved the database of Coffin *et al.* (*36*) by refining province ages and polygon outlines using recently published studies (dataset S3). We also now incorporate provinces that are up to 750 Ma old [e.g., (*18*); to access the database and find information on individual changes, see datasets S3 and S4, section S3, and fig. S2]. Similarly, we also again exclude any location where subsequent magmatism occurred within 500 km of the eruption site after 30 Ma of the initial eruption so that only the final phase of intraplate magmatism is included in our database (dataset S4). We again sample $z_{\mathrm{LAB}}^{1}$ within each polygon at 0.1° × 0.1° intervals.

**Fig. 2.**
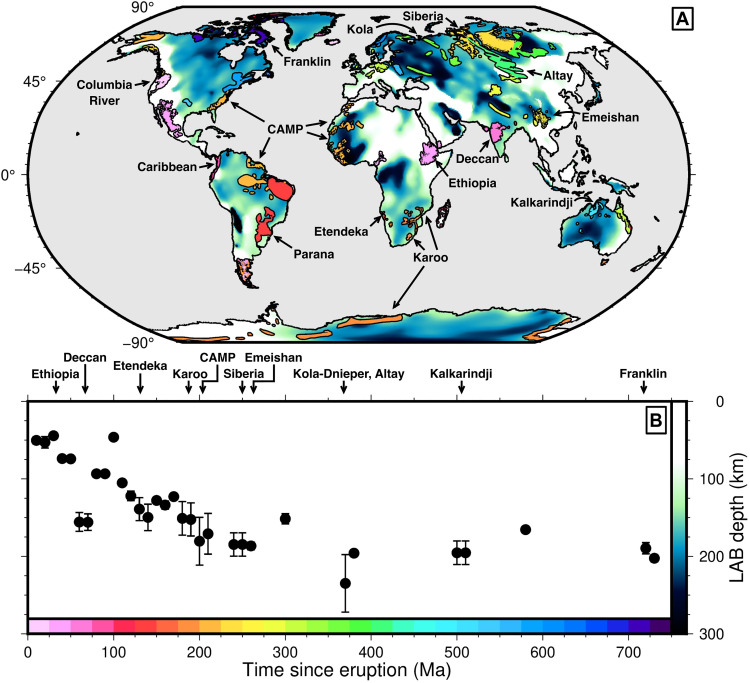
Global distribution of continental LIPs. (**A**) Distribution of continental LIPs colored by time since their eruption, overlain on map of continental LAB depth (*8*). Scale bars located on axes of (B); labels indicate names of LIPs mentioned in the main text. (**B**) Modern-day LAB depth beneath LIPs as a function of time since eruption. Labels indicate age of LIPs mentioned in the main text.

Figure 2 shows depth to the LAB beneath continental LIPs as a function of time since their emplacement [i.e., $z_{\mathrm{LAB}}^{1}(t_{0})$]. For the first time, we reveal a striking relationship between LAB depth and time elapsed since continental LIP emplacement. For recent provinces, the lithosphere is ∼50 to 70 km thick, which is consistent with that observed in the oceans and a range of geochemical proxies for lithospheric thickness [i.e., estimates calculated by (*20*) and new estimates derived from major element thermobarometry]. LAB depth progressively increases as a function of time since LIP emplacement to approximately 195 km at 250 Ma before flattening off. For example, the Ethiopian Flood Basalts were erupted at *t*_0_ ∼ 30 Ma ago, with magmatism continuing to recent times, and are underlain by lithosphere that is <50 km thick (*19*, *20*). The Paraná-Etendeka Traps, where *t*_0_ ∼ 138 to 128 Ma ago, on the other hand, are underlain by lithosphere that is 100 to 150 km thick. However, 195-km-thick lithosphere underlies the Siberian Traps, which were erupted at an age of *t*_0_ ∼ 250 Ma. This thickness is similar to the LAB depth beneath the ∼510-Ma-old Kalkarindji LIP in central and northwestern Australia, and the ∼750− to 710-Ma-old Franklin LIP in the Canadian Arctic [e.g., see (*18*); section S2]. The Deccan Traps (age *t*_0_ ∼ 66 Ma) mark the only significant outlier to this relationship, having markedly thicker lithosphere than LIPs of a similar age. In general, and with reference to our oceanic analysis, this result hints at lithospheric rejuvenation before, or during, magmatism followed by relaxation after magmatism ceases. Significantly, the depth at which $z_{\mathrm{LAB}}^{1}$ stops increasing and flattens off (i.e., ∼195 km) is about 60 to 90 km deeper than that observed in the oceans (compare Fig. 1D, black circles, and Fig. 2B).

We acknowledge that lateral transport of lava flows or dykes may mean that we sample lithosphere that is far away from the eruption site. In section S4.1, we show the raw, unaveraged values of $z_{\mathrm{LAB}}^{1}$ as a function of eruption age. We further test our results by exploiting two alternative compilations of LIP eruption centers from Torsvik *et al.* (*40*) and a new compilation assembled for this study (see sections S4.2 and S4.3 and dataset S5). We also verify that this relationship is robust against a raft of alternative lithospheric thickness models and is consistent with gravity observations (section S4.2). Finally, it is important to note that our analysis encompasses LIPs erupted onto all types of continental environments, ranging from shields (e.g., eastern Siberian Traps) to Phanerozoic sediments (e.g., Central Atlantic Magmatic Province, i.e., CAMP, and Karoo).

### Thermal modeling

It is clear that intraplate magmatism occurs above thinner-than-expected lithosphere and that older magmatic provinces reside above progressively thicker lithosphere. Rifting, which leads to rapid thinning of the lithosphere (i.e., shallowing of the LAB), is usually followed by protracted time-dependent rethickening as a result of conductive cooling. Similarly, the systematic increase in LAB depth beneath magmatic provinces from young to old suggests that the plate thickens as a function of time following the cessation of magmatism. This deepening eventually stalls and flattens out at a constant thickness of around ∼195 km beneath continental provinces and ∼125 km in the oceans. Two principal models have been developed to explain time-dependent thickening of the lithospheric plate. First, a half-space model, in which the lithosphere cools and thickens indefinitely as a function of age, and second, a plate model, in which the lithosphere cools and thickens, initially closely matching the half-space model, before approaching a finite thickness controlled by the convective resupply of basal heat [e.g., (*10*, *41*)]. The impact of this heating is generally approximated by imposing a constant basal potential temperature, *T*_p_, at a particular depth, *z*_p_, that defines the maximum depth to which conductive cooling can penetrate (*23*, *28*). Note that *z*_p_ is not the same as *z*_LAB_. Here, we investigate whether conductive cooling, represented in this case by a plate model, can explain the time-dependent thickening of the lithosphere, *z*_LAB_(*t*), following intraplate magmatism.

#### Oceanic intraplate magmatic provinces

Again, we begin with the oceanic lithosphere. We model one-dimensional thermal evolution, *T*(*z*, *t*), beneath intraplate magmatic provinces and hence depth to the LAB as a function of time, *z*_LAB_(*t*), by making three simple modifications to the plate model of Richards *et al.* (*23*, *28*) (Materials and Methods). First, rather than assuming an initial temperature profile consistent with mid-ocean ridge conditions (i.e., LAB depth ∼10 km below sea level), a steady-state initial temperature profile with an LAB depth equal to 50 km is imposed so that $z_{\mathrm{LAB}}^{0}$ = 50 km when magmatism occurs. In practice, this constraint means initially placing the 1175°C isotherm at a depth of 50 km. This assumption is consistent with both the results of our geochemical modeling and tomographically constrained LAB depth beneath intraplate magmatic provinces. Second, we assume that the less conductive oceanic crustal layer is 20 km rather than 7 km thick, a choice that reflects elevated thickness beneath magmatic provinces relative to standard oceanic crust. Note that we ignore the effects of viscoelastic relaxation, which means that the topographic load generated by this thicker crust is instantaneously compensated. Finally, observational studies have strongly suggested that initial excess subsidence of ocean islands is generated by movement away from swell bathymetry that is at least partly generated by excess mantle temperatures (*38*). Hence, to account for the excess heat provided by a putative mantle plume, initial steady-state temperature profiles corresponding to elevated initial mantle potential temperature, (*T*_p_)_0_, are imposed. As the thermal structure evolves, (*T*_p_)_0_ decays linearly over time (30 Ma) to the ambient mantle value adopted by Richards *et al.* (*28*), *T*_p_ = 1333°C (see Materials and Methods). This evolving temperature excess is initially imposed at the base of the thermal boundary layer (i.e., the shallowest depth at which $\frac{dT}{dz}$ drops below 0.5°C km^−1^), before deepening at a rate of 10 mm year^−1^. This progressive temperature decay and vertical advection of the thermal boundary simulate the movement of the plate away from a plume-like mantle heat source (*38*). We test the effect of varying the decay time of this thermal anomaly and its precise parametrization, but it makes minimal difference to our results (see section S5). To find the optimum plate model that best describes our *z*_LAB_(*t*) observations, we carry out a grid search in which pairs of *z*_p_ and (*T*_p_)_0_ are systematically explored and optimized.

**Fig. 3.**
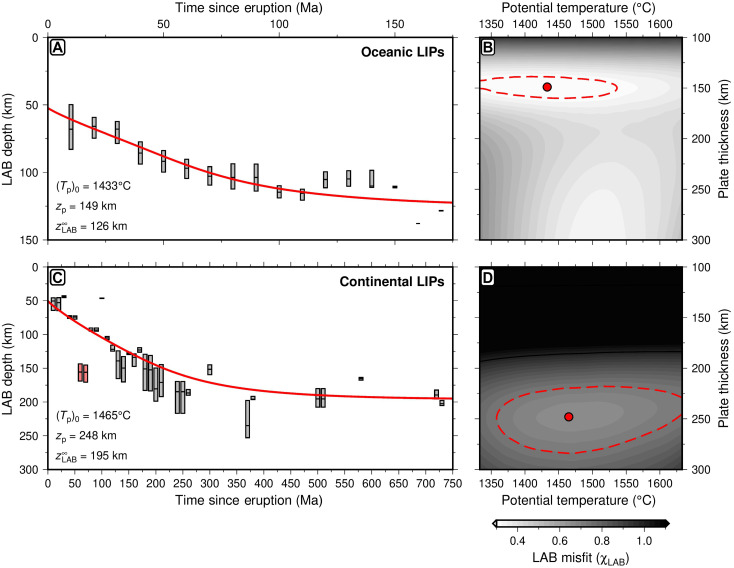
Lithospheric thermal modeling. (**A**) Observed and modeled LAB depth beneath oceanic intraplate magmatic provinces as a function of time since eruption. Gray bar: interquartile range of LAB depth distribution; red line: best-fitting modeled LAB depth. (*T*_p_)_0_: initial potential temperature; *z*_p_: plate thickness; $z_{\mathrm{LAB}}^{\infty }$: asymptotic depth to the LAB as *t* → ∞. (**B**) Misfit between observed and calculated LAB depth as a function of plate thickness, *z*_p_, and initial potential temperature, (*T*_p_)_0_, for oceanic intraplate magmatism (see Materials and Methods for details). Red circle: misfit minimum; red dashed line: contour marking 1.5× value at misfit minimum. Optimum values given in lower left-hand corner of (A). (**C**) Observed and modeled LAB depth beneath continental LIPs as a function of time since eruption. Red bars: Deccan Traps, excluded from the analysis. (**D**) As (B) but for continental LIPs. Optimum values of (*T*_P_)_0_, *z*_p_, and $z_{\mathrm{LAB}}^{\infty }$ given in lower left-hand corner of (C).

Our results show that cooling and rethickening of the lithosphere from an initial thickness of ∼50 km following the cessation of magmatism can explain the distribution of oceanic LAB depth as a function of time since magmatic eruption. The optimal plate thickness is closely comparable to that obtained by fitting global oceanic bathymetry and heat flow data as a function of plate age (*23*, *28*). The significant difference is that while the best-fitting value of *z*_p_ is only ∼17 ± 10 km in excess of that required to explain global oceanic subsidence and heat flow, an initial excess temperature anomaly of between 0 and +200°C relative to the global background is required to fit *z*_LAB_(*t*) beneath intraplate magmatic provinces [Fig. 3A; (*28*)]. This temperature anomaly then wanes rapidly. This result is consistent with both excess asthenospheric potential temperatures associated with at least some proportion of intraplate magmatism and constraints from the drowning of ocean islands (*20*, *38*, *39*).

#### Continental LIPs

Next, we extend this model to the continental realm. First, we maintain the condition that the initial LAB depth is 50 km (i.e., $z_{\mathrm{LAB}}^{0}$ = 50 km). This assumption is supported by modeling of REE compositions of mafic and ultramafic melts in LIP magmas (*39*). Second, we assume that continental crustal thickness, *z*_cc_ = 35 km, and account for differences in thermal properties associated with its more felsic composition including enhanced radiogenic heat production, H*, and higher thermal conductivity [see Materials and Methods; (*42*)]. As before, the initial temperature profile is determined by finding the steady-state geotherm, given the value of (*T_p_*)_0_, that results in the 1175°C isotherm at a depth of 50 km. We carry out a grid search to determine the best-fitting values of (*T*_p_)_0_ and *z*_p_ that are consistent with observed values of *z*_LAB_ as a function of LIP age. The Deccan Traps are excluded from our analysis since its two contributing temporal bins are clear outliers (see section S5).

Results show that, as for the oceans, the thickness of the lithospheric mantle beneath continental LIPs can be fitted with a simple adapted plate-cooling model, although with some significant differences. The best-fitting potential temperature is (*T*_p_)_0_ = 1465 ± 120°C, which, while less well defined, overlaps with the range of values of (*T*_p_)_0_ in the oceanic realm (Fig. 3). These results suggest that significantly elevated mantle potential temperatures [i.e., (*T*_p_)_0_ > 1345°C] are a systematic requirement of LIP formation, providing independent evidence for their formation above a mantle plume (*39*, *43*). However, the optimal plate thickness, *z*_p_ = 248 km, is significantly greater than in the oceans, which implies a steady-state LAB depth of $z_{\mathrm{LAB}}^{\infty }$ ≈ 195 km. This depth aligns more closely with lithospheric thicknesses inferred from subsidence in intra-cratonic basins (i.e., ∼200 km) rather than either subsidence observed in rift basins on non-cratonic continental lithosphere or the ocean floor. [i.e., ∼100 to 150 km; (*10*, *23*, *44*, *45*)]. Note that, although we have chosen to exclude the Deccan Traps outliers from our analysis, the best-fitting value of *z*_p_ is minimally affected by their inclusion (i.e., an increase of 1 km), while optimal (*T*_p_)_0_ remains ∼80°C above ambient values (i.e., 1413°C versus 1333°C; see section S5.1).

In summary, our analysis, in which we fit LAB depth estimates with conductive cooling models, suggests that the lithospheric mantle is rapidly thinned immediately before and/or during magmatism in both oceanic and continental realms. It then cools and progressively rethickens following magmatism. For continental LIPs, this thinning is associated with a significant temperature anomaly and steady-state plate thicknesses are similar to those in intra-cratonic basins.

## DISCUSSION

Our global analysis of lithospheric thickness beneath intraplate magmatic provinces has shown that magmatism occurs exclusively on thin lithosphere. Following eruption, the lithospheric mantle progressively cools and rethickens to a depth of 125 km in the oceans and 195 km on the continents. These inferences are supported by a range of observations. First, major and trace elemental compositions of recent intraplate magmas suggest that magmatism occurs only where the lithosphere is less than 80 km thick. Second, the thickness of the lithospheric plate calculated by exploiting seismic tomographic models and a calibrated *V*_S_-to-*T* conversion scheme is in broad agreement with these geochemical estimates. Third, the thickness of the lithosphere beneath ancient intraplate magmatic provinces is systematically thinner than would be expected if they were randomly distributed atop oceanic lithosphere that was following the well-documented plate-cooling relationship. Finally, lithospheric thickness defined by seismic tomographic imaging beneath both oceanic and continental intraplate magmatic provinces systematically increases as a function of eruptive age following a plate cooling relationship subject to initially elevated asthenospheric temperatures. A key observation is that several continental LIPs overlie cratons, which are regions of supposedly ancient, thick, and stable lithospheric mantle (e.g., Karoo, CAMP, Emeishan, Siberia, Kola-Dnieper, Kalkarindji, and Franklin). Our results suggest that even cratonic continental interiors are significantly less stable than often assumed, with cratonic roots apparently neither able to resist being destroyed during LIP emplacement nor remaining permanently thinned after the cessation of magmatism. Here, we discuss the implications of the relationship between LIP emplacement, rapid lithospheric thinning, and protracted thermal relaxation for the compositional makeup of continental lithosphere through time, the formation of economic resources, and the initiation of mass extinction events.

### Recratonization

Previous insights indicate that thick, cratonic lithosphere is likely to have been generated by high degrees of melt extraction at shallow depths followed by lithospheric thickening during ancient (i.e., pre-Phanerozoic) orogenic events [see (*15*, *46*) and references therein]. Our results, however, suggest that in some locations, this preexisting lithospheric mantle is destroyed before, or during, LIP eruption. It is then reemplaced over the subsequent ∼300 Ma by thermal relaxation (Figs. 2B and 3C). Compositional differences between unperturbed and rethickened cratonic lithosphere are challenging to distinguish geophysically. However, our results allow useful constraints to be placed upon the compositional evolution of cratonic roots during and after magmatism.

**Fig. 4.**
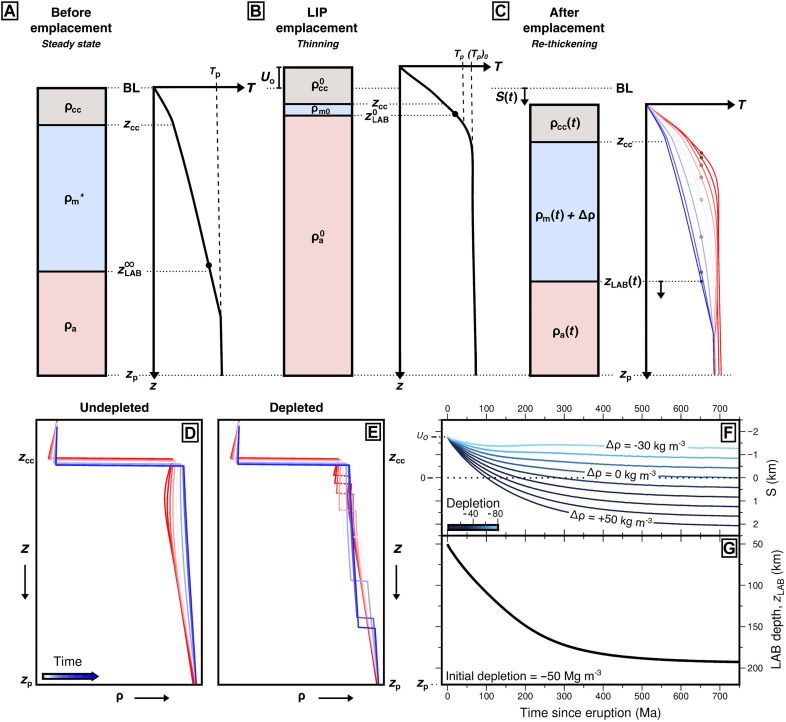
Thermal, depletion, and subsidence modeling. (**A**) Pair of cartoons showing continental column and temperature profile of precursory lithosphere in thermal steady state before LIP emplacement. $z_{\mathrm{LAB}}^{\infty }$: steady-state LAB depth, defined by 1175°C isotherm; *z*_cc_: crustal thickness; *z*_p_: plate thickness; ρ_cc_: steady-state crustal density; $\rho_{m}^{*}$: steady-state initial lithospheric mantle density; ρ_a_: steady-state asthenospheric density; *T*_p_: ambient mantle potential temperature; BL: base level (i.e., initial elevation of top of lithospheric column). (**B**) Thinned lithospheric column during LIP emplacement at *t* = 0, where $z_{\mathrm{LAB}}^{0}$ = 50 km; *U*_o_: initial uplift driven by thinning of plate and subplate thermal anomaly; $\rho_{m}^{0}$: lithospheric mantle density; $\rho_{a}^{0}$: asthenospheric mantle density; (*T*_p_)_0_: elevated mantle potential temperature. (**C**) Subsidence as *z*_LAB_(*t*) deepens from $z_{\mathrm{LAB}}^{0}$ to $z_{\mathrm{LAB}}^{\infty }$. ρ_cc_(*t*): crustal density as a function of time; ρ_m_(*t*): lithospheric mantle density as a function of time; ρ_a_(*t*): asthenospheric mantle density as a function of time; Δρ: density difference as a result of depletion between precursor, $\rho_{m}^{*}$, and final lithospheric mantle density, $\rho_{m}^{\infty }$; *S*(*t*): time-dependent net subsidence (i.e., above or below base level), *S*(∞) = 0 km if Δρ = 0 Mg m^−3^. (**D**) Density as a function of depth for undepleted lithospheric mantle colored by time since eruption (i.e., red to blue through time). (**E**) As (D) with depleted lithospheric mantle, Δρ = −50 kg m^−3^. (**F**) Air-loaded subsidence as a function of time since eruption, *S*(*t*), for various values of Δρ after thinning precursory lithosphere with initial depletion = −50 kg m^−3^ from $z_{\mathrm{LAB}}^{\infty }$ = 200 km to $z_{\mathrm{LAB}}^{0}$ = 50 km. Negative values indicate uplift. *U*_o_ = 1.76 km is transient initial uplift. Note if rethickening lithosphere more depleted than original lithosphere net uplift generated when *t* → ∞. Dotted line: no net uplift or subsidence. Labeled lines colored by depletion relative to ambient (undepleted) mantle. Labels: values of Δρ. Negative values are density reductions due to increased depletion. (**G**) Depth to LAB as a function of time since eruption, *z*_LAB_(*t*).

LIPs often appear on ancient cratons (e.g., Siberia, Karoo, Central Atlantic Magmatic Province, and Franklin), which are likely to have depleted, low-density lithospheric roots (*16*). Thinning of undepleted lithospheric mantle is a significant means of generating topographic uplift and denudation on 10^3^ km length scales (*47*). Conversely, thickening of an undepleted lithospheric root drives time-dependent subsidence (*48*). Lithospheric depletion (i.e., density reduction, Δρ) modulates the amplitude of this uplift and subsidence. Consider a cratonic setting where the onset of magmatism is associated with a combination of a transient asthenospheric thermal anomaly and removal of an ancient, thermally equilibrated, and depleted (i.e., low-density) cratonic root (Fig. 4, A and B). In this case, the initial density of the root, $\rho_{m}^{*}$, is perhaps 50 kg m^−3^ lower than in non-cratonic lithospheric mantle. Removal of this somewhat depleted root and the subplate thermal anomaly together lead to significant amounts of air-loaded surface uplift [i.e., *U*_o_ = 1.8 km if (*T*_p_)_0_ = 1465°C; see Materials and Methods]. Note that if $\rho_{m}^{*}$ is significantly more depleted, then the magnitude of this uplift decreases substantially (e.g., *U*_o_ ≈ 500 m for depletion of 80 kg m^−3^). Subsequent thermal reequilibration drives regrowth of the lithospheric mantle (Fig. 4, C to G). If this regrowing lithospheric mantle is completely undepleted, then the result is net air-loaded subsidence of 1.6 km after 350 Ma and 2.1 km after 750 Ma. No net uplift or subsidence occurs when rethickening mantle lithosphere is depleted by 50 kg m^−3^ (i.e., same density as precursor lithospheric mantle, $\rho_{m}^{*}$). Finally, if depletion is increased to 80 kg m^−3^, then a total of 1.3 km of net uplift is generated after 750 Ma (Fig. 4, F and G; Materials and Methods).

Extensive evidence exists for uplift associated with intraplate magmatism, although in cratonic regions it can be controversial [e.g., (*20*, *43*, *49*–*51*)]. This controversy perhaps indicates the relatively modest magnitude of initial uplift expected as a result of rapid thinning of depleted cratonic lithosphere. This more modest magnitude is in contrast to the kilometer-scale uplift expected to result from removing the same thickness of fertile lithosphere [e.g., (*47*, *52*, *53*)]. A second important observation is that many LIPs that overlie reequilibrated lithosphere (i.e., t_0_ > 300 Ma) are exposed at the surface today and not buried under deep sedimentary basins. It is therefore likely that they have experienced either net uplift or at least no appreciable net vertical motion between the pre-magmatism and post-equilibration lithospheric states. Together with the results of our simple modeling, these observations suggest that depleted cratonic lithosphere is removed before or during magmatism and then it is replaced by similarly or more depleted lithospheric mantle during subsequent thermal relaxation. LIP emplacement therefore provides a mechanism for cratonic destruction followed by recratonization.

Additional evidence corroborates this conclusion. There are two reasons to expect that the crust may sometimes be thinned during the emplacement of an LIP, which would be expected to generate even further net subsidence after lithospheric reequilibration (i.e., when *z*_LAB_ reaches $z_{\mathrm{LAB}}^{\infty }$). First, the initial asthenospheric thermal pulse and lithospheric removal generate excess topography that is progressively eroded, resulting in crustal thinning (*54*). Second, many—but not all—LIPs are associated with some amount of lithospheric extension. These processes are offset to some degree by the addition of erupted, intruded, and underplated basaltic material that would tend to thicken the crust (*54*). It is important to note, however, that all that remains of many LIPs is the plumbing system, the flood basalts having been totally denuded [e.g., (*18*, *55*)]. To expose LIP plumbing systems at the surface rather than have them buried under kilometers of sediment and lava flows, post-LIP topography must acquire permanent subcrustal support. Lithospheric depletion, potentially modulated by magmatic underplating, is a plausible mechanism for this topographic support. An important corollary is that initial uplift, erosion, and replacement of depleted lithospheric mantle with a subsequently less depleted (i.e., denser, in this case) lithospheric keel could be a way to generate subsidence in intra-cratonic basins.

This argument suggests that the emplacement of an LIP is likely responsible for local-to-regional destruction of cratonic lithospheric mantle on short time scales, followed by recratonization on longer time scales. There is a caveat worth highlighting, however. Our analysis provides neither an estimate for the precise thickness and composition of the preexisting lithospheric mantle beneath individual LIPs nor a definitive explanation for the mechanism of lithospheric mantle thinning. For example, it is possible that a mantle plume could be directed by basal lithospheric topography into a preexisting lithospheric thin spot [e.g., (*5*, *56*, *57*)]. There, it would generate surface uplift and extensive magmatism due to its excess potential temperature, before growth of a depleted, neutrally buoyant lithospheric root begins after magmatism, resulting in net-neutral topography. This situation may yield a similar suite of observations to those in the previously discussed case, where a neutrally buoyant cratonic root is first removed. However, independent constraints on paleo-lithospheric thickness from both the presence of kimberlites and the reconstruction of geothermal gradients using thermobarometry of mantle xenoliths indicate that the lithosphere was thick before several African magmatic events where it is now thinner [see, e.g., (*19*, *53*, *58*)]. Similar observations are also found in the MacKenzie Flood Basalt Province, Canada (*21*). Furthermore, our results themselves suggest that thin spots are short lived and heal in the absence of perturbation. Basal thermal erosion by a mantle plume has been proposed as a mechanism for thinning the lithospheric mantle, but this process is challenging to achieve on short time scales (*58*). Alternatively, melt and fluid infiltration could lead to refertilization and subsequent destabilization of the lithospheric keel (*15*, *59*, *60*).

Regardless of the precursory state of the mantle, simple geodynamic reasoning further supports rethickening of the lithospheric mantle after magmatism by progressive cooling of depleted restites. The equilibrium thickness of undepleted continental lithospheric mantle is around 100 to 150 km (*48*). We find that the equilibrium thickness of the thermal boundary layer in continental regions affected by LIP emplacement is closer to 180 to 220 km. McKenzie and Richter (*61*) showed that the thickness of the upper thermal boundary layer, δ, is proportional to $\nu^{\frac{1}{3}}$, where ν is kinematic viscosity (i.e., η/ρ, where η is dynamic viscosity and ρ is density). At a given temperature, melt depletion leads to an increase in viscosity of the mantle residue [e.g., (*62*)]. Hence, a doubling of ν leads to an increase in δ of around one quarter. Geochemical depletion of the uppermost mantle therefore leads to a thickening of the upper thermal boundary layer relative to ambient mantle.

Although time-dependent deepening of the LAB is a globally robust relationship, excess deepening is not observed in the oceanic realm, where $z_{\mathrm{LAB}}^{\infty }$ = 125 km. In the oceans, the LAB seldom exceeds depths of >150 km, regardless of the presence or absence of LIPs. Furthermore, in the oceans, significant post-magmatic subsidence is observed (*37*, *38*). A mechanism is therefore needed to rethicken depleted mantle on the continents while perhaps not in the oceans. Numerical modeling carried out by Liu *et al.* (*21*) indicates that lithospheric thin spots surrounded by thick cratonic lithosphere can trap accumulations of low-density, depleted mantle material. This material can be generated by in situ melt extraction within the plume head during LIP formation or can be composed of remnant parcels of previously removed cratonic mantle. Their results show that in these circumstances the lithosphere can recover up to 90% of its original thickness as depleted residue cools over the subsequent 300 Ma, almost entirely healing any putatively destroyed precursory cratonic lithospheric mantle. By contrast, their analysis implies that when LIPs are emplaced in oceanic regions distant from thick cratonic lithosphere, melt-depleted material is more easily entrained into the sublithospheric mantle, limiting the eventual rethickening of the oceanic lithosphere. Our observations strongly support this idea and suggest that, despite their rarity, where plumes are able to erode the cratonic lithospheric mantle, plume-driven recratonization is routine in the continental realm.

### Economic resources

Our results provide a predictive framework for modeling the thermal evolution of undeformed continental interiors in response to magmatic events. The thinning and rethickening pattern can act as a useful tool to highlight and evaluate prospects for economic resources that are dependent on the thermal evolution of the lithosphere, including critical minerals [e.g., (*8*)]. For example, diamonds primarily form within cold, thick continental lithospheric roots (i.e., 1130° ± 120°C at 5.3 ± 0.8 GPa) and are brought to the surface within kimberlite pipes (*63*). Our results suggest that thinning of the plate during/before emplacement of an LIP will suppress diamond formation and entrainment for ∼300 Ma as the plate rethickens beyond ⪆170 km (Fig. 2B). Several LIPs are spatially proximal to older, diamondiferous and/or younger, barren kimberlite fields. Such locations include the Siberian Traps, which erupted 75 to 45 Ma after the diamondiferous Alakit, Upper Muna, and Daldyn fields and 0 to 30 Ma before the barren, traps-related Kharamay field (*64*). The Keweenawan LIP on the southern Superior craton, North America, erupted 1150 to 1100 Ma before present, and preceded/coincided with the barren 1100-Ma-old Kyle Lake kimberlite field. Crucially, however, the much more recent Attawapiskat kimberlites, which occurred in the same location 180 to 150 Ma before present, were diamondiferous (*65*). Similar logic applies to the Artemisia kimberlites, which became diamondiferous 600 Ma after emplacement of the MacKenzie Flood Basalts above the Slave Craton, Canada (*21*). Previous authors have tied these relationships to the heating of lithospheric roots out of the diamond-bearing window by mantle plumes during LIP events (*19*, *66*, *67*). We contend that rapid and wholesale lithospheric removal may be a key mechanism (*19*). Moreover, our observations reveal a predictive time scale over which cratonic keels will return to a state favorable to diamond formation, which is similar to results of previous modeling studies [∼300 Ma, (*21*)].

### Climate change and mass extinctions

Finally, our results have implications beyond the solid Earth since LIP eruptions release large quantities of chemical species that can lead to global environmental change. When carbon, sulfur, and halogens are expelled into the atmosphere, they warm the planet, cool the planet, and deplete the ozone layer, respectively (*68*). The rapid and large-scale eruption of these climate-forcing gases during LIP emplacement is thought to be responsible for many of Earth’s mass extinctions [see, e.g., (*69*) and references therein]. The Siberian Traps (∼1 × 10^6^ to 2 × 10^6^ km^3^) coincides with the largest known extinction event, which defines the Permian-Triassic boundary and represents the loss of >80% of marine species (*70*). However, conventional mantle plume-derived melts are insufficiently concentrated in C, S, and halogens to generate the catastrophic environmental change required to drive such a mass extinction (*3*, *68*). Metasomatized continental lithospheric mantle, on the other hand, is rich in these elements (*71*). Analysis of mantle xenoliths emplaced during and after eruption of the Siberian Traps demonstrates that the subcontinental lithospheric mantle stored abundant halogens, ∼70% of which were scavenged by ascending melts (*71*). Therefore, destruction of the subcontinental lithospheric mantle and release of the volatiles it contains during LIP formation is a viable trigger for the Permian-Triassic boundary extinction, and was probably enhanced by release of S and C from sediments as a result of magmatic heating (*71*–*73*). While it has been previously argued that destruction of the lithospheric mantle beneath the very largest LIPs contributes to the most significant mass extinction events, a temporal, if not mechanistic, link has also been drawn between mass extinctions and smaller LIPs. For example, the Emeishan Traps, which are around ∼3 × 10^5^ km^3^ in volume (i.e., <1/3 Siberian Traps), and erupted above the Yangtze Craton, have been linked to the end-Guadalupian mass extinction event (*74*). Alteration and removal of the lithospheric mantle beneath even the smaller LIPs could therefore be a contributing factor to the environmental forcing that leads to mass extinction events.

## MATERIALS AND METHODS

### LIP database

Coffin and Eldholm (*35*) and Coffin *et al.* (*36*) mapped the outlines of Phanerozoic LIPs and oceanic seamounts, and provided approximate eruption ages for each location. We have updated this database to include LIPs up to 750 Ma. We further amended the ages and outlines of a number of polygons contained in the original database to honor updated radiometric dates. A list of sources for the updated database can be found in the Supplementary Materials (datasets S3 and S5).

### Seismologic estimates of lithospheric thickness

Throughout this study, we exploit the lithospheric thickness model of Hoggard *et al.* (*8*). This model converts shear-wave velocities, *V*_S_, into temperature, *T*, using the calibration method of Richards *et al.* (*24*) and the upper-mantle *V*_S_ tomographic model of Schaeffer and Lebedev (*75*). This temperature conversion scheme relies on the experiment-based parameterization of Yamauchi and Takei (*76*), which accounts for the effects of anelasticity on *V*_S_ as the mantle melting temperature is approached. The equations include seven material constants that are calibrated by exploiting the thermal structure beneath mid-oceanic ridges in four different ways. First, *V*_S_ profiles are extracted along oceanic plate flow lines and are averaged to yield *V*_S_ as a function of plate age and depth. These stacked profiles are then compared to predicted values of *V*_S_ from applying the *V*_S_-to-*T* conversion to a plate-cooling model (*23*). Misfit is calculated between observed and modeled *V*_S_ at depth slices of 87.5 and 112.5 km. Since oceanic plate thickness is generally ≤125 km, it is not possible to use this approach at greater depths. Second, *V*_S_ profiles are averaged globally at depths beneath the thermal boundary layer (i.e., between 225 and 400 km) and are compared to values of *V*_S_ predicted by applying the *V*_S_-to-*T* conversion to a mantle isentrope with a potential temperature, *T*_p_ = 1333°C, which is assumed to represent ambient mantle. Third, the globally averaged shear wave attenuation as a function of depth beneath old (>100 Ma) oceanic lithosphere is calculated from *V*_S_ using the same conversion and compared to published estimates at depths >150 km. Finally, the misfit between published and calculated depth-averaged mantle viscosity beneath the thermal boundary layer is evaluated.

These four misfit functions are combined, weighted, and minimized to optimize the values of the seven unknown material constants in the anelastic parameterization. Finally, lithospheric thickness is calculated globally by extracting the 1175°C isotherm, which is assumed to be a proxy for the depth to the base of the mechanical boundary layer. For further details on the methodology, see Hoggard *et al.* (*8*) and Richards *et al.* (*24*).

### Geochemical estimates of lithospheric thickness

#### Major element thermobarometry

Partitioning of major elements between melt and solid mantle is pressure and temperature dependent. The Plank and Forsyth (*25*) thermobarometer exploits a comprehensive suite of melt equilibration experiments to parameterize the response of major element composition to pressure and temperature changes. To estimate lithospheric thickness, we apply this thermobarometer to a global database of Neogene-Quaternary mafic intraplate magmatic samples compiled by Ball *et al.* (*20*). These calculations are carried out using meltPT—an open-source Python library designed for whole-rock thermobarometric analysis [https://github.com/fmcnab/meltPT; (*26*)].

Before estimating melt equilibration conditions, the effects of fractional crystallization are mitigated by filtering our magmatic database so that all samples have 9 < MgO < 14.5 wt %. Samples must also include a measurement of Ce concentration so that H_2_O can be estimated assuming that H_2_O/Ce = 200 (*77*, *78*). We calculate the primary melt compositions of each sample by adding olivine that is in equilibrium with the melt until olivine forsterite content is 0.9 (*79*). Since only Fe^2+^ is compatible within olivine, an estimate of Fe^3+^/ΣFe is required. We assume that Fe^3+^/ΣFe = 0.2, which is halfway between the average values for theoliitic and alkali basalts (*80*).

These primary melt compositions are used to generate estimates of the pressure and temperature at which mantle melts equilibrated [i.e., *P*_eq_ and *T*_eq_, respectively (*25*)]. Samples may have last equilibrated within the lithosphere or asthenosphere, and the boundary between these layers is defined throughout our study by the 1175°C isotherm. In a given location, we assume that the maximum lithospheric thickness coincides with the equilibration pressure of the shallowest sample that has an equilibration temperature *T*_eq_ > 1175°C since temperatures cooler than this value are likely to indicate that equilibration occurred within the lithosphere. To estimate lithospheric thickness globally, we subdivide our thermobarometric results into 1° × 1° geographic bins. We only estimate lithospheric thickness for bins with ≥10 samples since smaller sample sizes are less likely to capture the LAB.

#### REE inverse modeling

We exploit the results of REE inverse modeling presented by Ball *et al.* (*20*), who use an adapted form of the INVMEL-v12 forward model to simulate partitioning of trace elements between melt and residue within the mantle during melting (*81*). REE concentrations along adiabatic melt paths are calculated, and misfit between observed and synthetic REE concentrations is minimized using a grid search procedure. Ball *et al.* (*20*) collect their data into 1° × 1° geographic bins and calculate an optimum melt path for each bin. The upper limit of the melting region is assumed to represent the LAB (*20*). Note that this definition is slightly different to the isothermal definition used elsewhere in our study. The partitioning of each REE depends upon the bulk source composition, the aluminous phase present in the source, as well pressure and temperature. Ball *et al.* (*20*) filter their database for 8.5 < MgO < 14.5 wt % to mitigate fractionation effects, and they use ɛNd as a proxy for source composition. Finally, they place the garnet-spinel transition zone at 63 to 72 km (*82*).

### Statistical analyses

#### Weighted median

To mitigate skewness and suppress outliers, we calculate the median when averaging data. For ease of analysis, we have divided Earth’s surface into 1° × 1° bins. To avoid oversampling higher latitudes and biasing median values, we weight each lithospheric thickness data point, *z_i_*, by latitude, ϕ*_i_*. To achieve this weighting, we construct a data vector, **z**, and a weights vector, **w**, where each weight is given by cosϕ*_i_*. The weighted median is calculated by sorting **z** and **w** in ascending order by the values of **z**. The median value is given by *z_j_*, where the index, *j*, of the sorted data vector is calculated by minimizing

$$j=\underset{k}{\min}\left[\sum_{i=1}^{k}w_{i}z_{i}>\frac{1}{2}\sum_{i=1}^{n}w_{i}z_{i}\right]$$ (1)

For even-length data vectors, we use the upper-weighted median so that *z_j_* is given by (*z_j_* + *z*_*j*+1_)/2. We calculate the weighted interquartile range of the distribution by changing the prefactor of $\frac{1}{2}$ in Eq. 1 to $\frac{1}{4}$ and $\frac{3}{4}$ for upper and lower quartiles, respectively.

#### Predicted oceanic lithospheric thickness distribution

Seamounts can only form on oceanic crust that is the same age, or older, than themselves. For example, a suite of seamounts erupting at the present day (i.e., 0 Ma) can cap oceanic crust that formed between the present day and Jurassic times. The median age of the seafloor beneath these seamounts will therefore lie somewhere between the two and be skewed toward younger ages since there is much more young oceanic crust than old on Earth (Fig. 1A). Moreover, a seamount that is 200 Ma can only be located on crust ≥200 Ma, most of which has been subducted and lost from the surface record. Consequently, since lithospheric plate thickness increases with age, we expect to observe a positive relationship between seamount age and LAB depth.

Richards *et al.* (*23*, *24*) constructed an oceanic plate-cooling model that simultaneously minimizes the misfit between global databases of heat flow and bathymetric observations that have been corrected for sediment and crustal loading. Their approach includes both pressure- and temperature-dependent thermal properties. We assume that the LAB within this plate-cooling model coincides with the 1175°C isotherm [i.e., consistent with the LAB model of Hoggard *et al.* (*8*)]. We predict the unperturbed depth to the modern-day LAB, $z_{\mathrm{LAB}}^{1}$, by extracting LAB depth as a function of age from this plate model using the oceanic crustal age model of Müller *et al.* (*27*), which was updated to include additional data by Richards *et al.* (*23*). The predicted value is compared to thickness implied by seismic tomography, which is in close agreement [see Fig. 1C; (*8*, *75*)].

Oceanic crustal ages, *t*_crust_, and LAB depths range from 0 Ma to *t*_max_ and from *z*_LAB_(0) to *z*_LAB_(*t*_max_), respectively. Seamounts cannot form on plates younger than their eruption age, *t*_0_, and if *t*_0_ > *t*_max_, then they would not be recorded on Earth’s surface today. Therefore, the thickness of lithosphere on which seamounts are located ranges from *z*_LAB_(*t*_0_) to *z*_LAB_(*t*_max_). For each eruption age, we record the lithospheric thickness distribution of oceanic crustal ages where *t*_0_ < *t*_crust_ < *t*_max_. The median and interquartile range of predicted lithospheric thickness as a function of *t*_0_ (i.e., the blue line in Fig. 3D) is calculated using Eq. 1.

### Lithospheric modeling

#### Parameterization

To model lithospheric rethickening following periods of active magmatism, we solve the one-dimensional heat equation expressed as

$$\rho (P,T,X)C_{P}(T,X)\frac{\partial T}{\partial t}=\frac{\partial }{\partial z}\left[k(P,T,X)\frac{\partial T}{\partial z}\right]+H^{*}(X)$$ (2)

where *t* is the time, *z* is the depth, *T* is the temperature, *P* is the pressure, *X* is the composition, ρ is the density, *C*_P_ is the isobaric specific heat capacity, *k* is the thermal conductivity, and H* is the internal radiogenic heat production.

Equation 2 is solved numerically with an unconditionally stable time- and space-centered Crank-Nicholson finite difference scheme and a predictor-corrector step (*83*). Accordingly, Eq. 2 is recast as

$$\begin{array}{c} T_{j}^{n+1}+A\left[-\frac{k_{j+\frac{1}{2}}^{m}}{\text{Δ}z_{j}^{m}}T_{j+1}^{n+1}+\left(\frac{k_{j+\frac{1}{2}}^{m}}{\text{Δ}z_{j}^{m}}+\frac{k_{j-\frac{1}{2}}^{m}}{\text{Δ}z_{j-1}^{m}}\right)T_{j}^{n+1}-\frac{k_{j-\frac{1}{2}}^{m}}{\text{Δ}z_{j-1}^{m}}T_{j-1}^{n+1}\right]\\ =T_{j}^{n}+A\left[\frac{k_{j+\frac{1}{2}}^{m}}{\text{Δ}z_{j}^{m}}T_{j+1}^{n}-\left(\frac{k_{j+\frac{1}{2}}^{m}}{\text{Δ}z_{j}^{m}}+\frac{k_{j-\frac{1}{2}}^{m}}{\text{Δ}z_{j-1}^{m}}\right)T_{j}^{n}+\frac{k_{j-\frac{1}{2}}^{m}}{\text{Δ}z_{j-1}^{m}}T_{j-1}^{n}\right]+{AH}_{j}^{*m}(\text{Δ}z_{j}^{m}+\text{Δ}z_{j-1}^{m}) \end{array}$$ (3)

where

$$A=\frac{\text{Δ}t}{\left[\rho_{j}^{m}C_{P\;j}^{m}(\text{Δ}z_{j}^{m}+\text{Δ}z_{j-1}^{m})\right]}$$ (4)

and Δ*t* is the time step, Δ*z* is the depth spacing between nodes, and *n* and *j* are the time and depth indices, respectively. Equation 3 is solved by tridiagonal elimination (*83*). For the initial predictor phase of each time step, *m* = *n*, while in the subsequent corrector phase, $m=n+\frac{1}{2}$. We use a Lagrangian reference frame, whereby ${\text{Δ}z}_{j}^{m}$ is initially set to 1 km (i.e., when *m* = 0) and then scales with thermal contraction in subsequent time steps. These time steps are calculated using a Courant-Friedrichs-Lewy condition calculated according to

$$\text{Δ}t=\min_{j}\left[\frac{{\left(\text{Δ}z_{j}^{0}\right)}^{2}\rho_{j}^{0}C_{P\;j}^{0}}{2.2k_{j}^{0}}\right]∼5\;\mathrm{kyr}$$ (5)

*T*^*n*+1^ typically converges to within a tolerance of 0.001°C after the corrector phase.

#### Boundary conditions

All models consist of crustal and mantle layers. Crustal thickness for oceanic and continental regions is set to *z*_oc_ = 20 km and *z*_cc_ = 35 km, respectively. In both cases, the underlying mantle extends from the Moho to an assumed equilibrium plate thickness, *z*_p_. The initial depth to the LAB, after the cessation of magmatic activity and lithospheric thinning, is assumed to be $z_{\mathrm{LAB}}^{0}$ = 50 km. To account for the possible presence of a mantle plume beneath the plate, initial asthensopheric potential temperature, (*T*_p_)_0_, can vary between 1333°C, which is assumed to be the temperature of ambient mantle, and 1633°C (i.e., an excess temperature of 300°C).

Separate parameterizations are used to define the thermophysical properties of the crust and mantle (*k*, *C*_P_, ρ, and H*). Crustal radiative thermal conductivity and ρ are determined using the Richards *et al.* (*28*) parameterization, but the reference density, ρ_0_, is dependent on whether oceanic or continental lithosphere is to be modeled (2950 and 2700 kg m^−3^, respectively). In the continental crust, *C*_P_ and lattice thermal diffusivity, κ_lat_, are calculated using the parameterization of Whittington *et al.* (*42*), and H* is assumed to be 0.7 μW m^−3^ (*8*). In oceanic crust, we use the Richards *et al.* (*28*) parameterization for *C*_P_ and κ_lat_, and assume H* = 0 mW m^−3^. In both the continental and oceanic mantle, the temperature- and pressure-dependent formulations specified in Richards *et al.* (*28*) are adopted, with radiogenic heat production assumed to be negligible (H* = 0 μW m^−3^).

Initial temperature profiles are obtained by combining the parameters outlined above with the equations of McKenzie *et al.* (*84*). In all cases, kinematic viscosity, ν, is assumed to be 9 × 10^19^ m^2^ s^−1^. For each combination of *z*_oc_ or *z*_cc_, and (*T*_p_)_0_, we find the steady-state geotherm consistent with $z_{\mathrm{LAB}}^{0}$ by iterating through a range of mechanical boundary layer thicknesses (2 to 60 km). We select the temperature profile with depth to the 1175°C isotherm equal to $z_{\mathrm{LAB}}^{0}$.

To simulate the waning of a plume-derived heat source through time, we impose an evolving basal boundary condition. At *t* = 0, *T*(*z*) is given by the initial isentrope defined by (*T*_p_)_0_ below the base of the thermal boundary layer (i.e., the shallowest depth at which the geothermal gradient, $\frac{\partial T}{\partial z}$, drops below 0.5°C km^−1^). In later time steps, the depth at which this boundary condition is imposed, *z*_b_, increases according to a prescribed plume sinking rate, *v*_plume_ = 10 mm year^−1^, until the deepest model node (i.e., *z*_b_ = *z*_p_) is reached, whereupon the basal boundary depth remains fixed. Simultaneously, from *t* = 0 to 30 Ma, the temperature applied at the basal boundary decays linearly to that of the ambient mantle isentrope (i.e., *T*_p_ = 1333°C) at the relevant depth. Beyond *t* = 30 Ma, the basal temperature is assumed to remain equal to that of the ambient mantle isentrope at the appropriate depth.

#### Optimization strategy

We carry out a two-parameter sweep to find the combination of plate thickness, *z*_p_, and initial potential temperature, (*T*_p_)_0_, that best explains our constraints on *z*_LAB_(*t*). For each [*z*_p_, (*T*_p_)_0_] pair, we find the initial steady-state thermal structure that yields $z_{\mathrm{LAB}}^{0}$ = 50 km. Note that, if the lithospheric mantle is instantaneously thinned, it is likely that the remaining mantle will not be thermally equilibrated. Nonetheless, since the conductive layer is thin, it will rapidly reach steady state and so the effect of thermal disequilibration is likely to be minor. We then calculate *z*_LAB_(*t*), for 0 < *t* < *t*_max_ Ma, where *t*_max_ = 750 Ma, using Eq. 3. To minimize the misfit between tomographically determined LAB depth and predicted LAB depth as a function of age beneath LIPs, we use the trial function

$$\chi =\sqrt{\frac{1}{M}\sum_{i=1}^{M}{\left(\frac{z_{i}^{o}-z_{i}^{c}}{\sigma_{i}}\right)}^{2}}$$ (6)

where $z_{i}^{o}$ and $z_{i}^{c}$ are observed and calculated LAB depth, σ*_i_* is the standard deviation of the *i*th measurement, and *M* is the number of estimates. We used weighted binned median lithospheric thickness estimates, where

$$\sigma_{i}=\frac{\sqrt{{\left({\mathrm{IQR}}_{1}^{i}\right)}^{2}+{\left({\mathrm{IQR}}_{2}\right)}^{2}}}{1.349}$$ (7)

and ${\mathrm{IQR}}_{1}^{i}$ is the interquartile range of the *i*th measurement and IQR_2_ = 25 km is the approximate vertical uncertainty of the lithospheric thickness grid. Note that this relationship assumes that errors are normally distributed.

#### Subsidence

Air-loaded subsidence of the surface for our pressure- and temperature-dependent plate model is given by

$$S(t)=\int_{0}^{z_{p}}\left[1-\frac{\rho (0,z^{\prime })}{\rho (t,z^{\prime })}\right]{dz}^{\prime }$$ (8)

where *z*′ is the Lagrangian depth coordinate that deepens as the column is compressed. Since many ancient LIPs are located above continental shields, we assume that the pre-LIP lithospheric mantle (i.e., where *z*_cc_ < *z* < *z*_LAB_) was in thermal equilibrium, cratonic, and that precursory lithospheric mantle density, $\rho_{m}^{*}$, is depleted by −50 Mg m^−3^ relative to ambient mantle (i.e., 3280 Mg m^−3^ at surface temperature and pressure relative to an ambient value of 3330 Mg m^−3^; Fig. 4A). The effect of changes in lithospheric depletion following LIP emplacement is explored by adding Δρ (i.e., Δρ is negative for more depleted lithosphere) to all values of ρ(*t*, *z*′) that are located at depths within the lithospheric mantle. For simplicity, we assume that the steady-state thermal structure of the lithospheric column before thinning and after rethickening are identical and given by the results of our thermal evolution modeling (i.e., $z_{\mathrm{LAB}}^{\infty }$; Fig. 3D). We explore initial geotherms that are out of thermal equilibrium in the Supplementary Materials. Initial uplift, *U*_o_ = *S*(0), after thinning is calculated using Eq. 8; thence, *S*(*t*) is calculated using a range of values of Δρ to explore the effect of lithospheric melt depletion upon subsidence of the Earth’s surface (Fig. 4).

## Supplementary Materials

### This PDF file includes:

Sections S1 to S5 Figs. S1 to S17 Legend for data files S1 to S6 References
