# Supplementary material for: Destruction and regrowth of lithospheric mantle beneath large igneous provinces
Source: Sci Adv. 2023 Sep 6;9(36):eadf6216. doi: 10.1126/sciadv.adf6216 (PMC11811892; doi:10.1126/sciadv.adf6216)
Supplement: Supplementary file 1 — Sections S1 to S5 Figs. S1 to S17 Legend for data files S1 to S6 References [file sciadv.adf6216_sm.pdf]

Science Advances
AAAS

# Supplementary Materials for

## Destruction and regrowth of lithospheric mantle beneath large igneous provinces

Simon N. Stephenson *et al.*

Corresponding author: Simon N. Stephenson, simon.stephenson@earth.ox.ac.uk

*Sci. Adv.* **9**, eadf6216 (2023)
DOI: 10.1126/sciadv.adf6216

**The PDF file includes:**

Sections S1 to S5
Figs. S1 to S17
Legend for data files S1 to S6
References

**Other Supplementary Material for this manuscript includes the following:**

Data files S1 to S6

# 1 Datasets

We provide a number of primary and ancillary datasets that we use in our main analysis. Extended data included in the Supplementary Materials to this publication include the following items. All data can also be found an open-source Zenodo repository (doi: 10.5281/zenodo.8169597).

1. Data S1. A database of global geochemical compositions of mafic intraplate magmatic rocks compiled by **Ball et al.** (*20*), and corresponding estimates of melt equilibration pressure and temperature ($P_{eq}$ and $T_{eq}$, respectively; this study). Note that authors should cite **Ball et al.** (*20*) in reference to the global geochemical database. They should cite **this study** in reference to the global equilibration pressure and temperature estimates, in which case they should also cite **M^cNab et al.** (*26*), whose software was used to calculate $P_{eq}$ and $T_{eq}$.

2. Data S2. A spreadsheet containing $z^1_{LAB}$ estimates beneath modern intraplate provinces. For complete references to geochemical analyses contained in this database, please see *Ball et al.* (*20*). The database includes lithospheric thickness estimates obtained

   (a) by exploiting melt equilibration pressure and temperature (this study);

   (b) by inverse modelling of rare earth element compositions (*20*); and

   (c) from the lithospheric thickness model of *Hoggard et al.* (*8*), which is based upon the tomographic model SL2013sv (*75*).

3. Data S3 & S4. A database containing outlines of magmatic provinces dating back to 750 Ma, including

   (a) a directory (Data_S3.zip) containing the unfiltered database shape files (lips.shp, lips.shx, lips.dbf, lips.cpg). This directory also contains the same data in a multisegment text file for plotting in the Generic Matting Tools (polys_ID_age_unfiltered.dat) in which each polygon is separated by '>' where the header indicates polygon ID and time since eruption. And

   (b) a database filtered for final magmatic event in a given location (Data_S4.dat), where each polygon header also contains '> ID age polygon_area'.

4. Data S5. A database of located LIP eruption centres (Data_S5.dat).

5. Data S6. A pdf document of references (Data_S6.pdf). The document includes

   (a) references used to update locations and ages of the large igneous province database of *Coffin et al.* [Dataset S3; (*36*)];

   (b) references for existing lithospheric thickness models used test our observed $z_{LAB}(t)$ relationship; and

   (c) references used to locate the eruption centres of mantle plumes (i.e. Dataset S5).

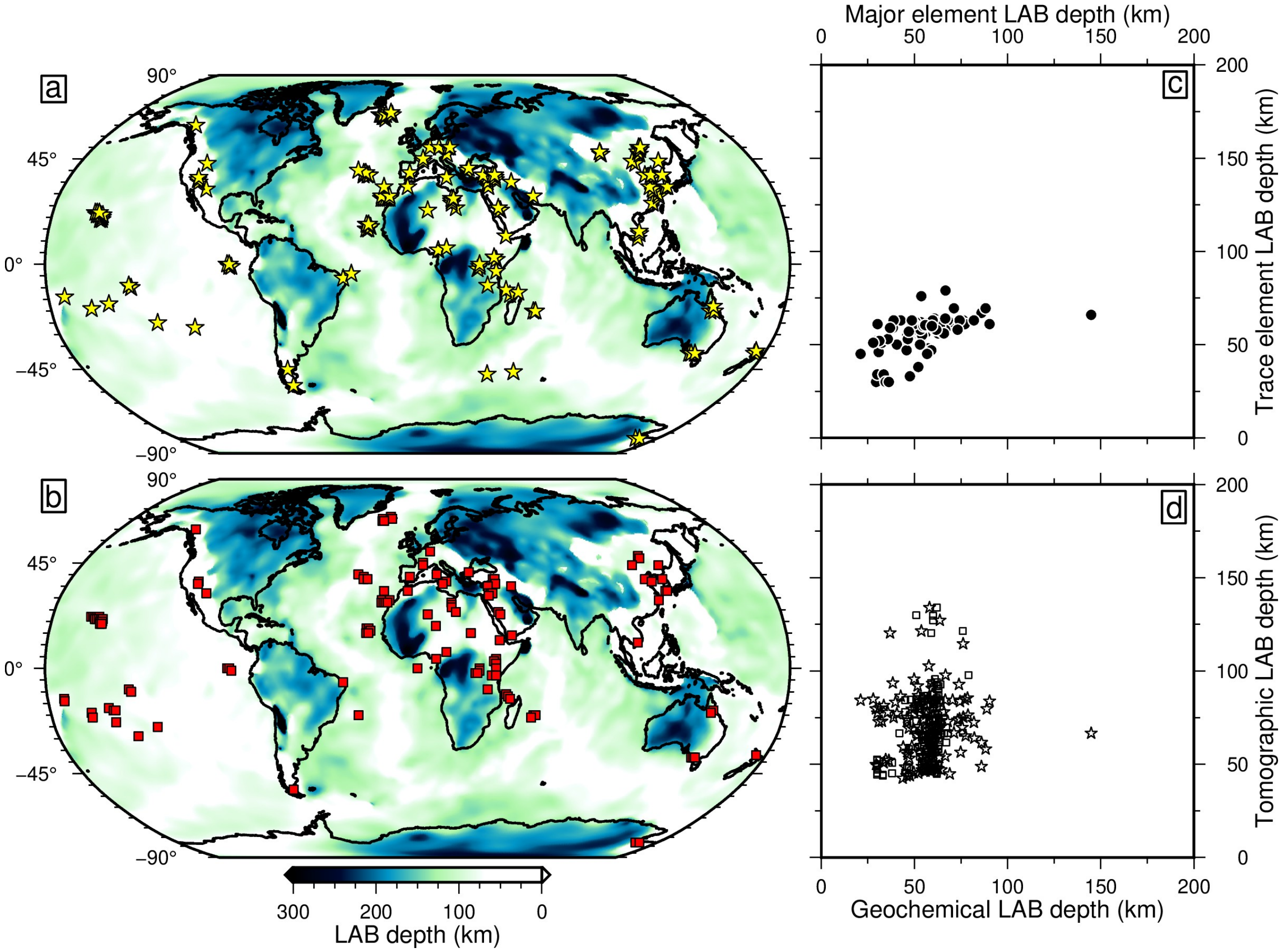


Figure S1: **Global distribution of modern-day igneous provinces with geochemically derived LAB estimates.** (a) LAB depth (*8*). Yellow stars = locations of LAB depth estimates obtained by finding magmatic equilibration pressure and temperature that corresponds to base of lithosphere (this study, see main text and Methods) (b) Same as panel (a). Red squares = locations of LAB depth estimates obtained by inverse modelling of rare earth element compositions of mafic rocks (*20*). (c) LAB depth determined by major element equilibration depth as a function of that determined by modelling rare earth element compositions. (d) Tomographically determined LAB depth as a function of geochemically-determined LAB depth. Symbol shapes correspond to symbols on panels (a) and (b).

## 2 Geochemical estimates of modern-day lithospheric thickness

The locations and values of present-day oceanic LAB depth estimates are shown in Figure 1 of the main text. These estimates come from three sources: (i) the tomographic lithospheric thickness model of *Hoggard et al.* (*8*), derived from the SL2013sv tomographic model of *Schaeffer & Lebedev* (*75*); (ii) inverse modeling of the rare earth element compositions of mafic igneous rocks (*20*); and (iii) major element thermobarometric modelling of sample compositions contained in the *Ball et al.* geochemical database [see Methods; (*20*)]. In Supplementary Figure S1a, we show the locations and corresponding LAB depth estimates for both the oceanic and continental realms. We exclude all provinces that are older than 10 Ma and/or have translated > 400 km from their eruption location (see ref. (*20*) for methodological details). For estimates determined using major element proportions to estimate equilibration pressures and temperatures, ∼ 98% of estimates lie on lithosphere < 80 km thick. For those determined by inverse modelling of rare earth element compositions, the corresponding value is 100%. On the other hand, ∼ 93% of tomographically-derived estimates are on lithosphere

thinner than 80 km (Supplementary Figure S1). All modern-day LAB depth estimates can be found in Database S2.

# 3 Large igneous province database

Note that references to the updates made to the database are found in the Data_S6.pdf file.

The starting point for our ocean island, seamount and plateau, and LIP databases is the digital map archive of ref. (*36*). In the oceans, we have supplemented this dataset with additional ocean islands, plateaux and seamounts, and excised polygons where radiometric dates are lacking and/or polygons cannot be tied to documented oceanic morphology (Figure S2). Our updates for the Arctic Regions include additions to Alpha Ridge and the Yermak Plateau (*86–88*). In the Atlantic Ocean, improvements have been made to the New England Seamounts, Rio Grande Rise, Vitória-Trinidade Ridge, and the Walvis Ridge (*89–92*). Indian Ocean edits include changes to the Kerguelan Plateaux, Ninetyeast Ridge, and 85°E Ridge (*93–95*). Additions to the Pacific Ocean include changes to the Austral-Cook Islands, the Boudeuse Ridge, the Hahajima seamount, Hawai'ian and Emperor Seamounts, the Line Islands, the Magellan Seamounts, the Marquesas archipelago, the Mid Pacific Mountains, the North Volcanic Ridge, the Pitcairn-Gambier mantle plume, the Society mantle plume, The Tavara seamounts, the Tuamotu Plateau, and the Western Pacific Seamounts (*38, 96–107*). Updates in the Southern Ocean are predominantly to the Agulhas Ridge (*108, 109*).

We have further improved this database by adding and/or updating the age range of a suite of continental LIPs using Figure 1 of *Bryan et al.* (*110*). These include: Sierra Madre Occidental (38 Ma), Whitsundays (120 Ma), Bunbury (132 Ma), Dronning Maud Land (182 Ma), Tasman (183 Ma), Chon Aike (188 Ma), Central Atlantic Magmatic Province (201 Ma), Angayuchan (210 Ma), Tarim (280 Ma), Panjal (289 Ma), European-northwest African Event (300 Ma), Kennedy-Connors-Auburn (310 Ma), Kola-Dneiper (370 Ma), Yakutsk (375 Ma), Kalkarindji (510 Ma), and Wichita (530 Ma). Altay-Sayan (400 Ma) was added using the Figures in *Vorontsov et al.* (*111*). Provinces 530–750 Ma, including Franklin/Thule (720 Ma), were added using the maps and accompanying references in *Ernst et al.* (*18*) with the exception of the Central Iapetus Magmatic Province (570–620 Ma) which was added using the data in *Youbi et al.* (*112*). Finally, The Keweenawan/Midcontinent Rift was removed since it was assigned an age of 620 Ma in the database of *Coffin et al.* (*36*), but is in actual fact around 1.1 Ga.

# 4 Lithospheric thickness - eruption age relationship

Note that references for the lithospheric thickness models cited in this Section are found in the Data_S6.pdf file.

## 4.1 Data distribution

In Figures 1 & 2 of the main text, we present the relationship between depth to the LAB and time since eruption of magmatic provinces by calculating a weighted moving median and interquartile range (see Methods). In Figure S3a, we show the raw relationship without calculating this moving average for both the oceanic and continental realms (see Database S4). Despite the spread in $z_{LAB}(t)$, there is a clear relationship between the depth to the LAB and the time elapsed since eruption. We also further show the unfiltered database (i.e. in this case without searching for the youngest volcanic episode in a given location; grey circles; see Database S3). Despite the unsurprising increase in scatter and greater number of datapoints plotting on thinner lithosphere where subsequent magmatism has occurred, it is clear that for both oceanic and continental lithosphere the lower limit of $z^1_{LAB}$ increases as a function of time since eruption. For young LIPs, the distribution of $z^1_{LAB}$ is concentrated at shallow depths, whereas for older provinces they extend onto progressively thicker lithosphere.

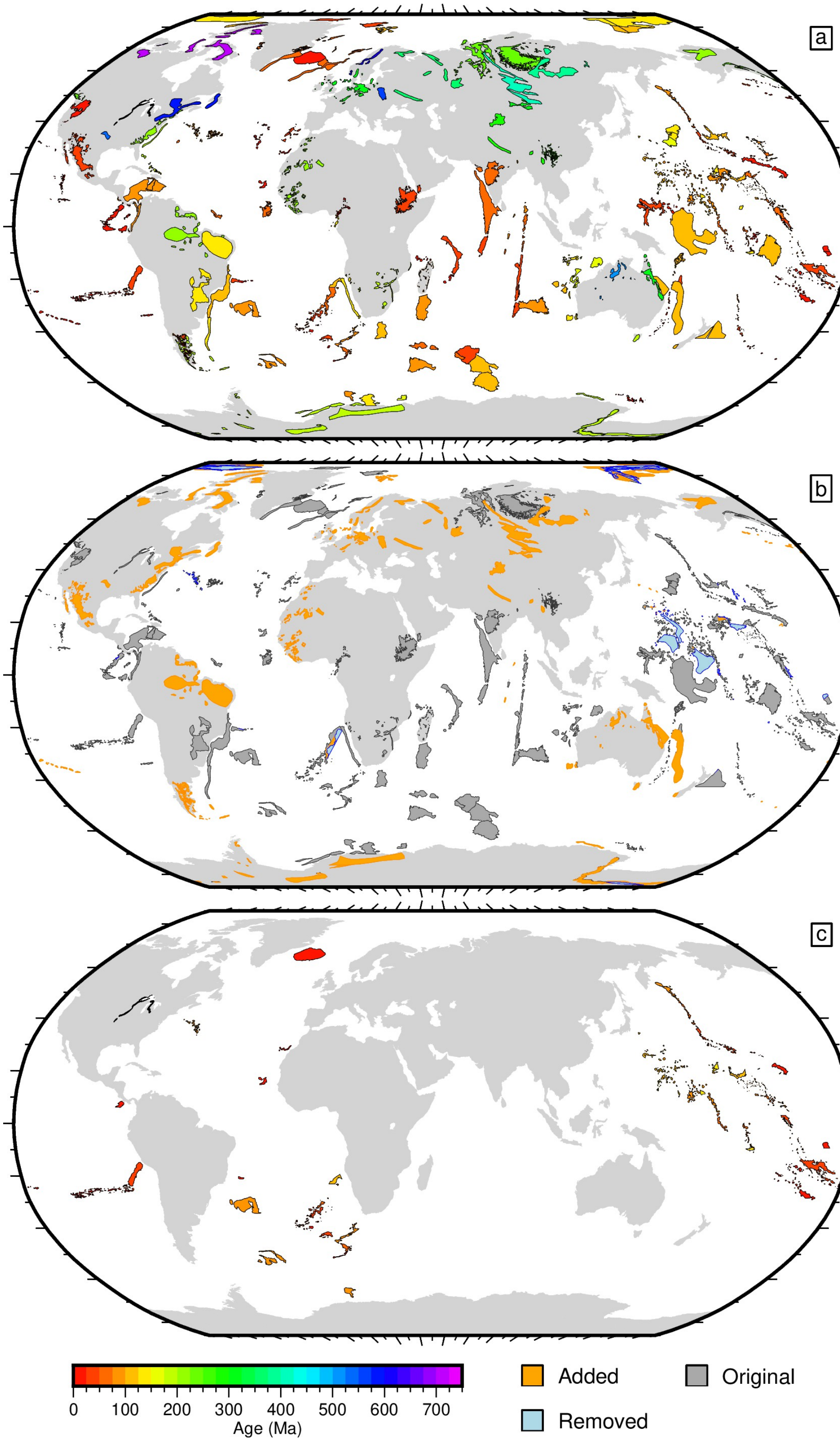


Figure S2: **Distribution of igneous provinces in space and time.** (a) Updated dataset in its entirety colored by eruption age. (b) Changes to the dataset compared to *Coffin et al.* (*36*); orange/blue/gray polygons = added/removed/original volcanic provinces. (c) Polygons in the original dataset updated to honor recent radiometric dating studies.

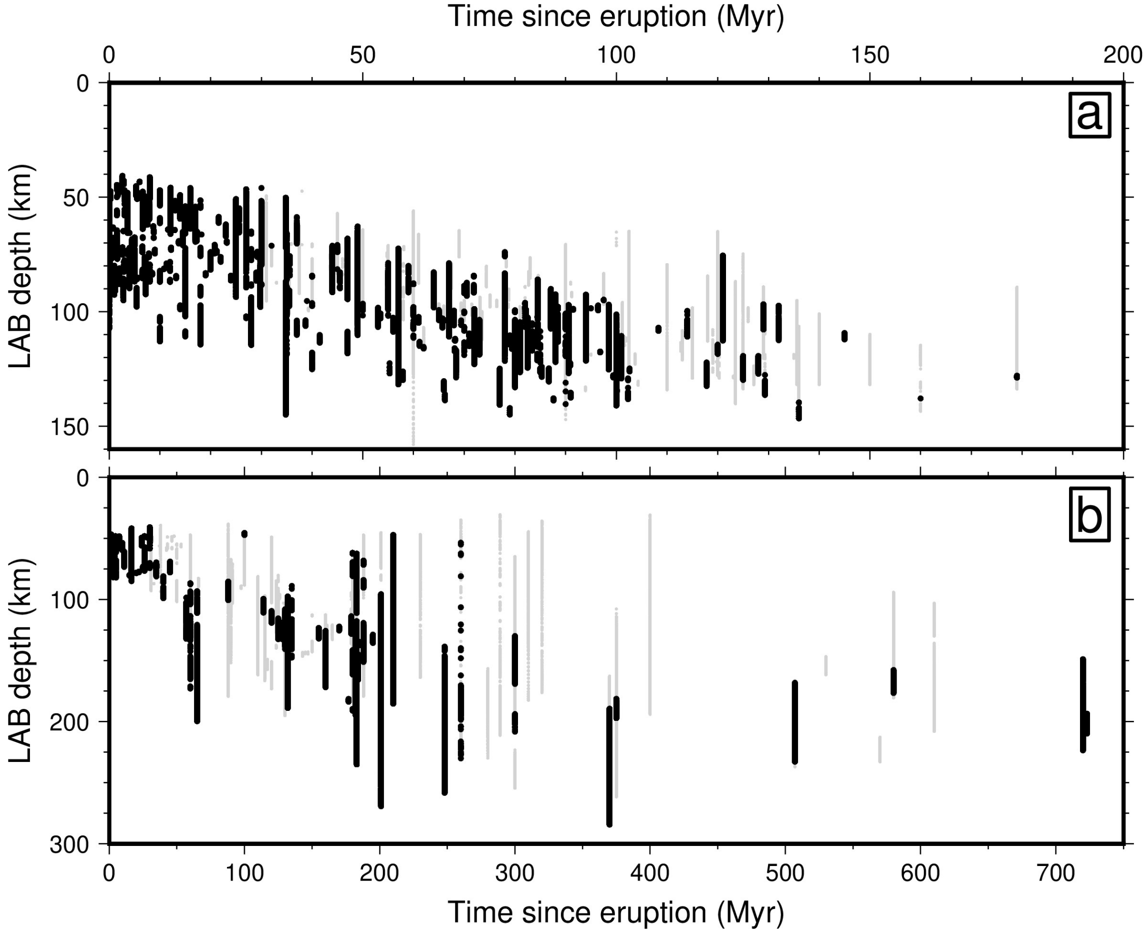


Figure S3: **Unbinned lithospheric thickness-magmatic eruption age relationship.** (a) Oceanic intraplate magmatic provinces. Black circles = individual $z^1_{LAB}$ estimates at $0.1 \times 0.1°$ bins within LIP polygons filtered according to methodology described in the main text; light gray circles = same as back circles but for unfiltered database. (b) Continental large igneous provinces. Note different *x*- and *y*-axis scales on (a) and (b).

## 4.2 Alternative lithospheric thickness models

We reproduce Figures 1 & 2 of the main text using a range of different shear wave tomographic models. *Hoggard et al.* (*8*) calculated depth to the 1175°C isotherm using the calibrated elastic-anelastic parameterisation of *Richards et al.* (*24*) using four different global tomographic shear-wave velocity models. We note that other approaches exist for estimating lithospheric thickness that exploit predominantly seismic tomography, heat flow and gravity measurements [see, e.g. refs (*113–115*)], but we have chosen to initially maintain consistency here since it best illustrates the differences between underlying tomographic models. Figures S4 and S5 show results if the SLNAAFSA model is used. SLNAAFSA is constructed by merging four shear wave tomographic models, incorporating the North American model of *Schaeffer et al.* (*116*), the South Atlantic model of *Celli et al.* (*117*), and the African model of *Celli et al.* (*19*), which are all blended into the global model of *Schaeffer & Lebedev* (*75*). Figures S6 and S7 are constructed using the 3D2015-07Sv shear wave velocity model of *Debayle et al.* (*118*). Figures S8 and S9 are based upon the the shear wave velocity model CAM2016 (*115, 119*). In all cases we use the results of *Hoggard et al.* (*8*) and not the lithospheric thickness values quoted within the tomographic study [e.g. compare ref. (*115*) and (*8*)]. It is important to note that each model is individually calibrated in order to mitigate the effects of smoothing and damping used in regularising the tomographic models (see Methods, ref. (*8*) and ref. (*28*) for more details). Previous work has demonstrated consistency between a range of tomographic models and independent geological observations, including mantle xenolith equilibration pressures and temperatures, oceanic residual depth anomalies, gravity anomalies, and the plate cooling relationship [e.g. refs (*8, 24, 51*)]. We note there are differences in detail but the main features are consistent between models, namely the increase in LAB depth as a function of magmatic province emplacement age, with a flattening of the relationship after 60–80 Ma in the oceanic, and ~ 250 Ma in the continental realms, respectively. These asymptotes correspond to a depth of of 100–130 km in the oceans and ~ 200 km on the continents. For eruption ages < 60–80 Ma, in the oceans, seamounts, islands and plateaux are associated with thinner than predicted lithosphere.

Next we test our results against two alternative, independent proxies for continental LAB depth. First we extract $z^1_{LAB}$ from the A2006 model of *Artemieva* [Figure S10; (*114*)]. This lithospheric thickness grid is based on heatflow estimates. We find that the relationship is much less clear than we see with a range of tomographically defined LABs. This discrepancy is not surprising, since heat flow data is patchily distributed and largely absent in many locations where LIPs are present (e.g. Siberia, Africa, South America, Australia). Finally, we investigate the relationship between the long-wavelength (i.e. > 700 km) free-air gravity anomaly and time since eruption (Figure S11). The long-wavelength gravity anomaly provides a crude proxy for the depth to the LAB, where negative gravity anomalies are associated with thick, cold lithospheric roots. Although scatter is significantly greater, there is a broad agreement between our results from tomographic models and the gravity anomaly. Generally recent LIPs are on +10-20 mGal anomalies, while older LIPs are on negative gravity anomalies, with the trend broadly flattening off between 0 to −20 mGal.

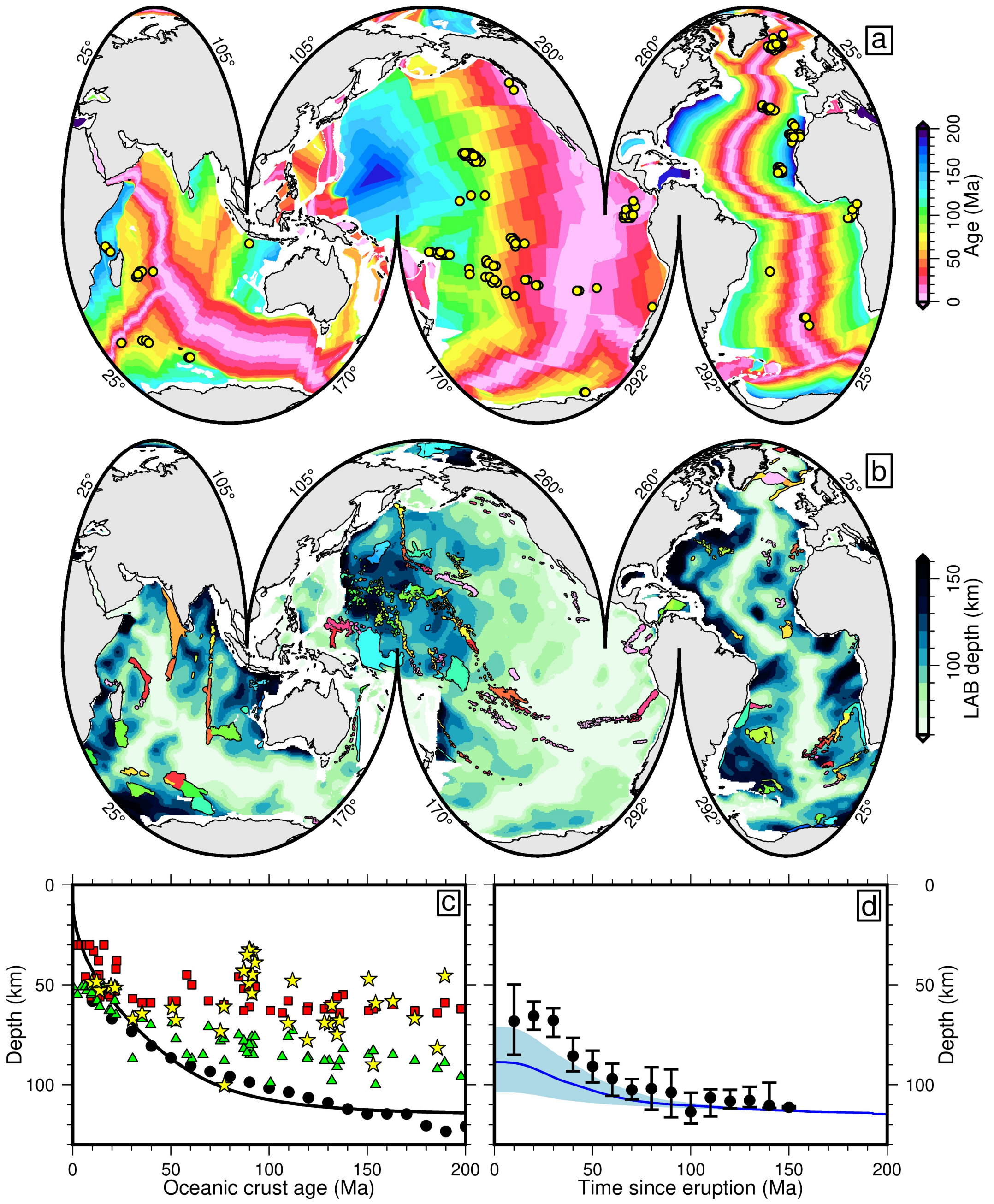


Figure S4: **Distribution of oceanic large igneous provinces exploiting** SLNAAFSA **tomographic model (*19, 75, 116, 117*).** (a) Oceanic plate age (*27, 85*). Yellow circles = locations of intraplate magmatic provinces (*20*). (b) Oceanic LAB depth. Coloured polygons = locations of oceanic magmatic provinces (i.e. ocean islands, seamounts and plateaux) coloured by age. (c) Constraints on modern-day LAB depth beneath intraplate magmatic provinces as a function of underlying oceanic crustal age. Red squares = LAB depth determined by inverse modelling rare earth element compositions of mafic rocks (*20*); green triangles = thickness determined by tomographic-derived LAB depth model shown in panel (b); yellow stars = LAB depth estimated by finding magmatic equilibration pressure and temperature that corresponds to base of lithosphere (this study, see main text & Methods). (d) Black circles with error bars = modern LAB depth beneath magmatic provinces as a function of province age; moving median at 10 Ma intervals with a ±20 Ma window; error bars = interquartile range; blue line = median expected distribution of LAB depth beneath LIPs if they were distributed randomly in time and space across oceanic lithosphere assuming plate model of Richards *et al.* (*24*) and plate age model of Muller *et al.* (*27*); blue envelope = interquartile range.

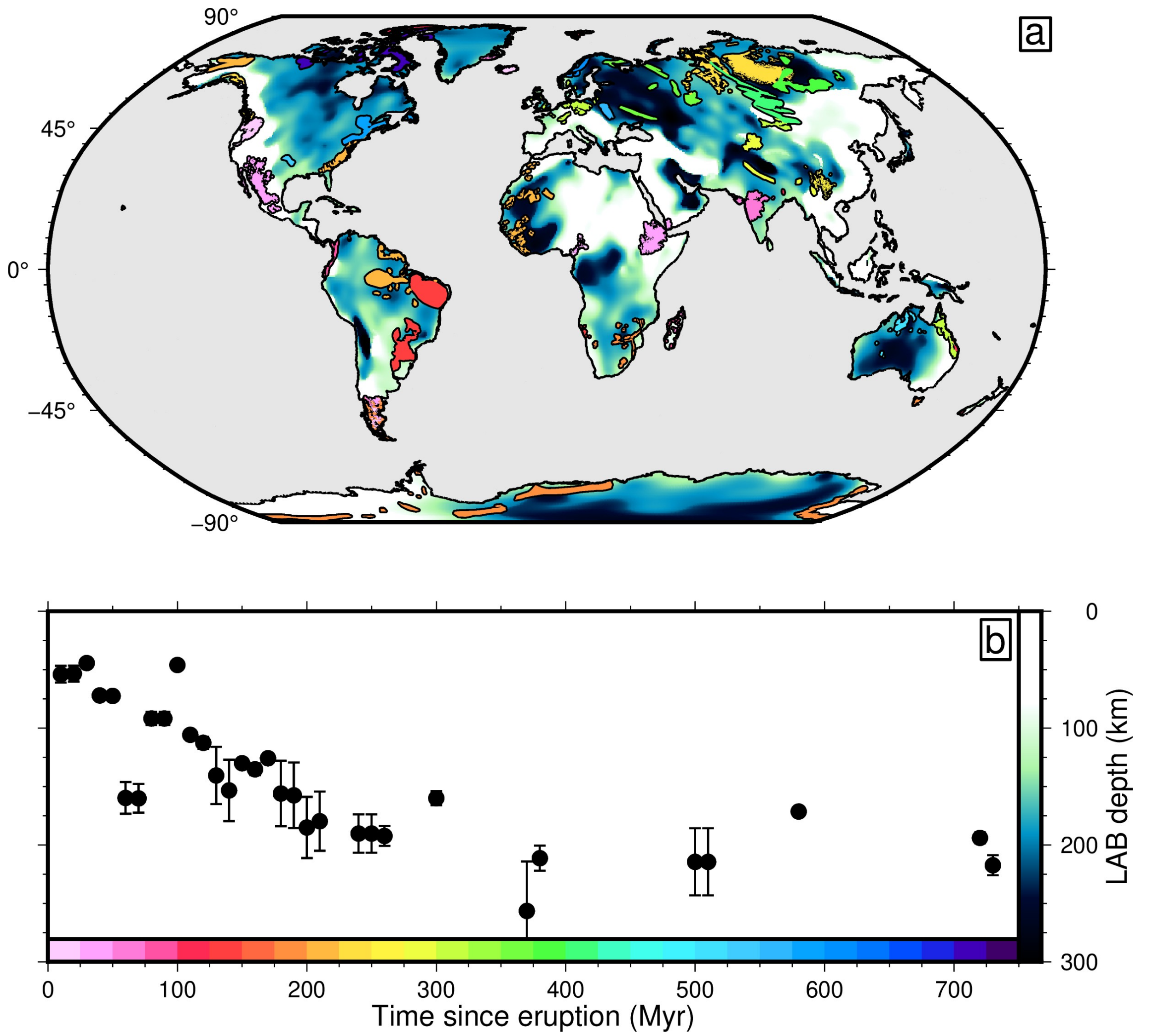


Figure S5: **Global distribution of continental large igneous provinces exploiting** SLNAAFSA **tomographic model (*19, 75, 116, 117*).** (a) SLNAAFSA model converted to lithospheric thickness (*8*). Polygons = distribution of continental large igneous provinces in space and time. Scale bars located on axes of panel (b). (b) Modern-day lithospheric thickness beneath large igneous provinces as a function of LIP age.

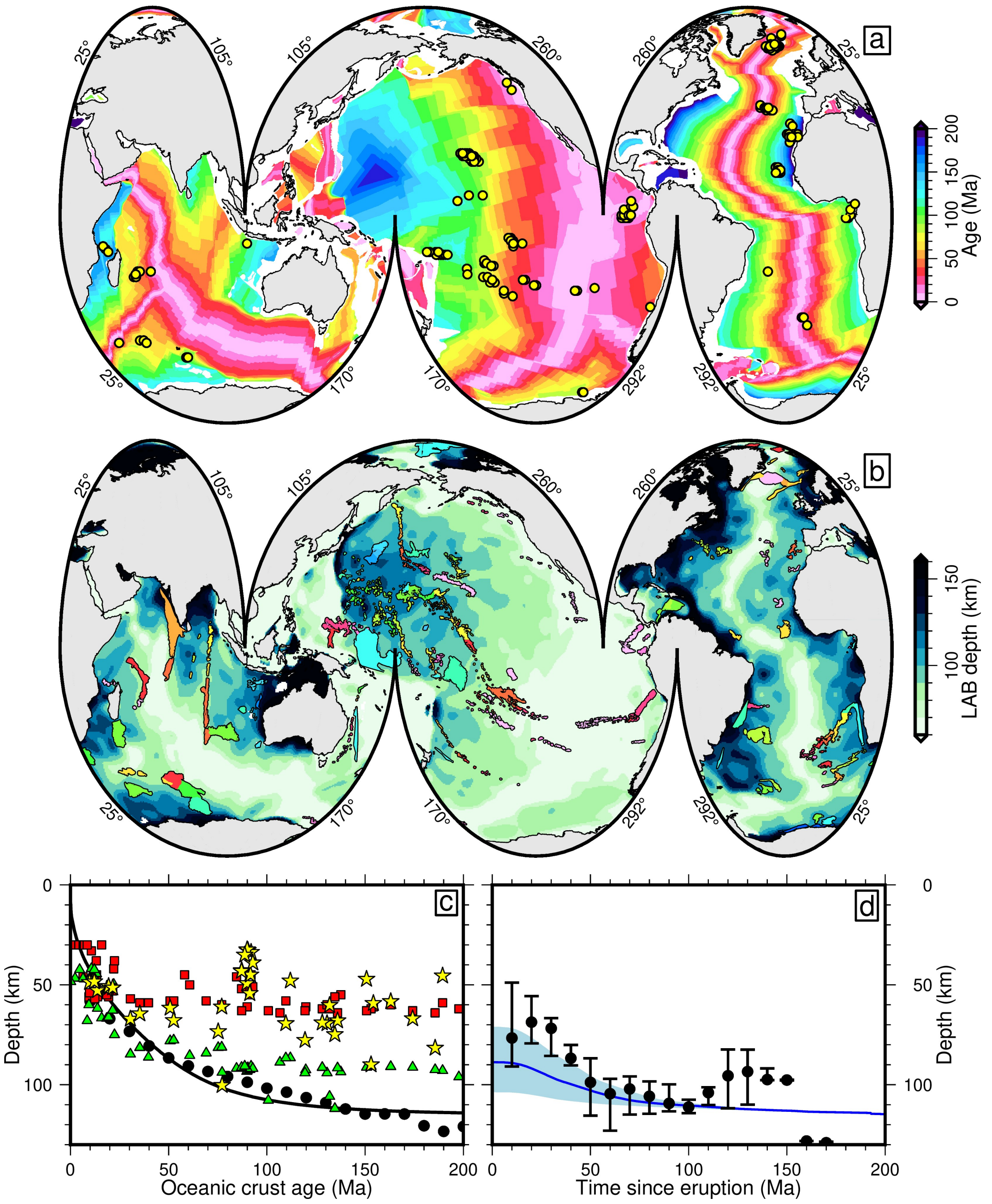


Figure S6: **Distribution of oceanic large igneous provinces exploiting** 3D2015-07Sv **tomographic model (*118*).** (a) Oceanic plate age (*27, 85*). Yellow circles = locations of intraplate magmatic provinces (*20*). (b) Oceanic LAB depth. Coloured polygons = locations of oceanic magmatic provinces (i.e. ocean islands, seamounts and plateaux) coloured by age. (c) Constraints on modern-day LAB depth beneath intraplate magmatic provinces as a function of underlying oceanic crustal age. Red squares = LAB depth determined by inverse modelling of rare earth element compositions of mafic rocks (*20*); green triangles = thickness determined by tomographic-derived LAB depth model shown in panel (b); yellow stars = LAB depth estimated by finding magmatic equilibration pressure and temperature that corresponds to base of lithosphere (this study, see main text & Methods). (d) Black circles with error bars = modern LAB depth beneath magmatic provinces as a function of province age; moving median at 10 Ma intervals with a ±20 Ma window; error bars = interquartile range; blue line = median expected distribution of LAB depth beneath LIPs if they were distributed randomly in time and space across oceanic lithosphere assuming plate model of Richards *et al.* (*24*) and plate age model of Muller *et al.* (*27*); blue envelope = interquartile range.

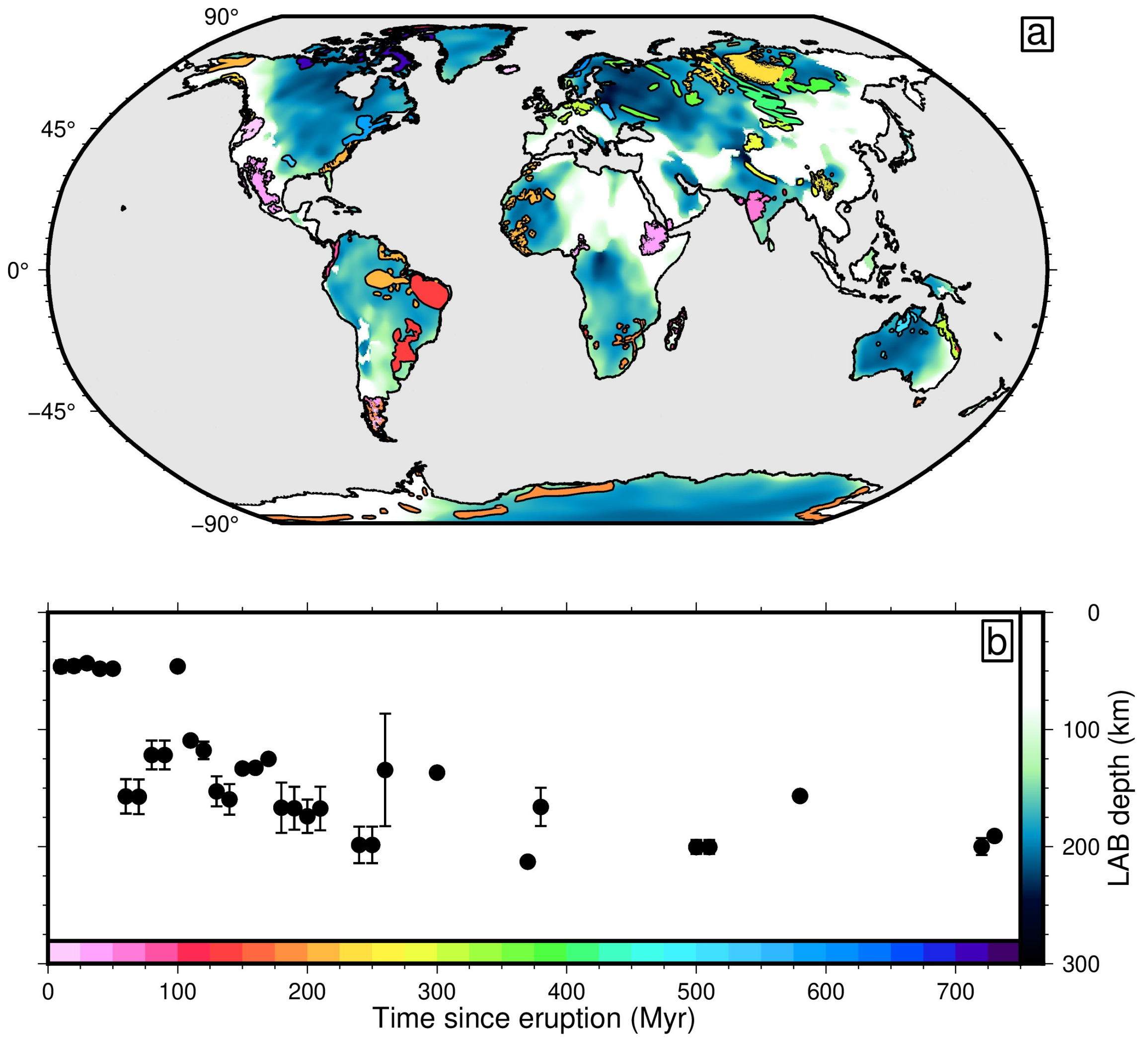


Figure S7: **Global distribution of continental large igneous provinces exploiting** 3D2015-07Sv **tomographic model (*118*).** (a) 3D2015-07Sv model converted to lithospheric thickness (*8*). Polygons = distribution of continental large igneous provinces in space and time. Scale bars located on axes of panel (b). (b) Modern-day lithospheric thickness beneath large igneous provinces as a function of LIP age.

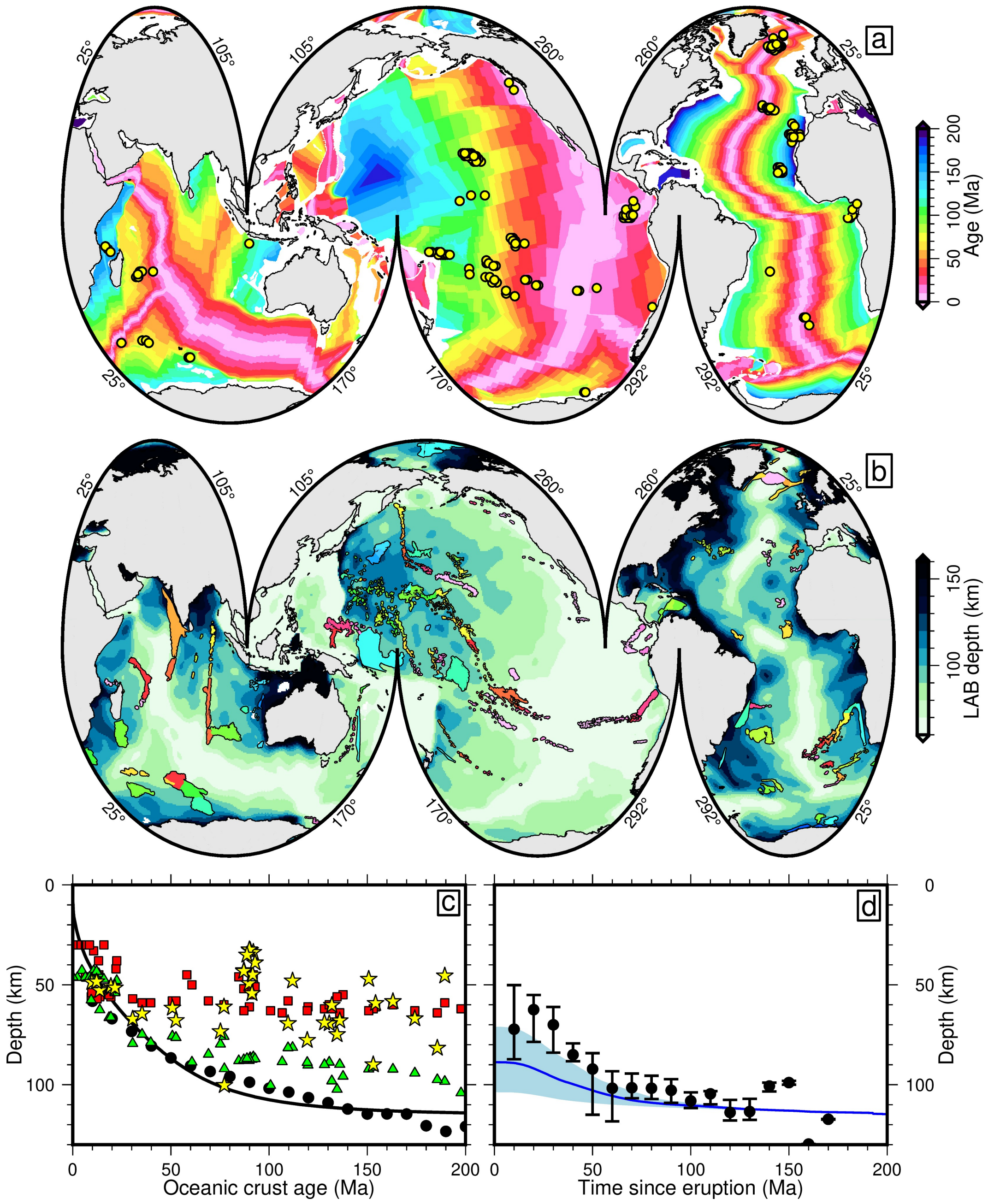


Figure S8: **Distribution of oceanic large igneous provinces exploiting** CAM2016 **tomographic model (*115*).** (a) Oceanic plate age (*27, 85*). Yellow circles = locations of intraplate magmatic provinces (*20*). (b) Oceanic LAB depth. Coloured polygons = locations of oceanic magmatic provinces (i.e. ocean islands, seamounts and plateaux) coloured by age. (c) Constraints on modern-day LAB depth beneath intraplate magmatic provinces as a function of underlying oceanic crustal age. Red squares = LAB depth determined by inverse modelling of rare earth element compositions of mafic rocks (*20*); green triangles = thickness determined by tomographic-derived LAB depth model shown in panel (b); yellow stars = LAB depth estimated by finding magmatic equilibration pressure and temperature that corresponds to base of lithosphere (this study, see main text & Methods). (d) Black circles with error bars = modern LAB depth beneath magmatic provinces as a function of province age; moving median at 10 Ma intervals with a ±20 Ma window; error bars = interquartile range; blue line = median expected distribution of LAB depth beneath LIPs if they were distributed randomly in time and space across oceanic lithosphere assuming plate model of Richards *et al.* (*24*) and plate age model of Muller *et al.* (*27*); blue envelope = interquartile range.

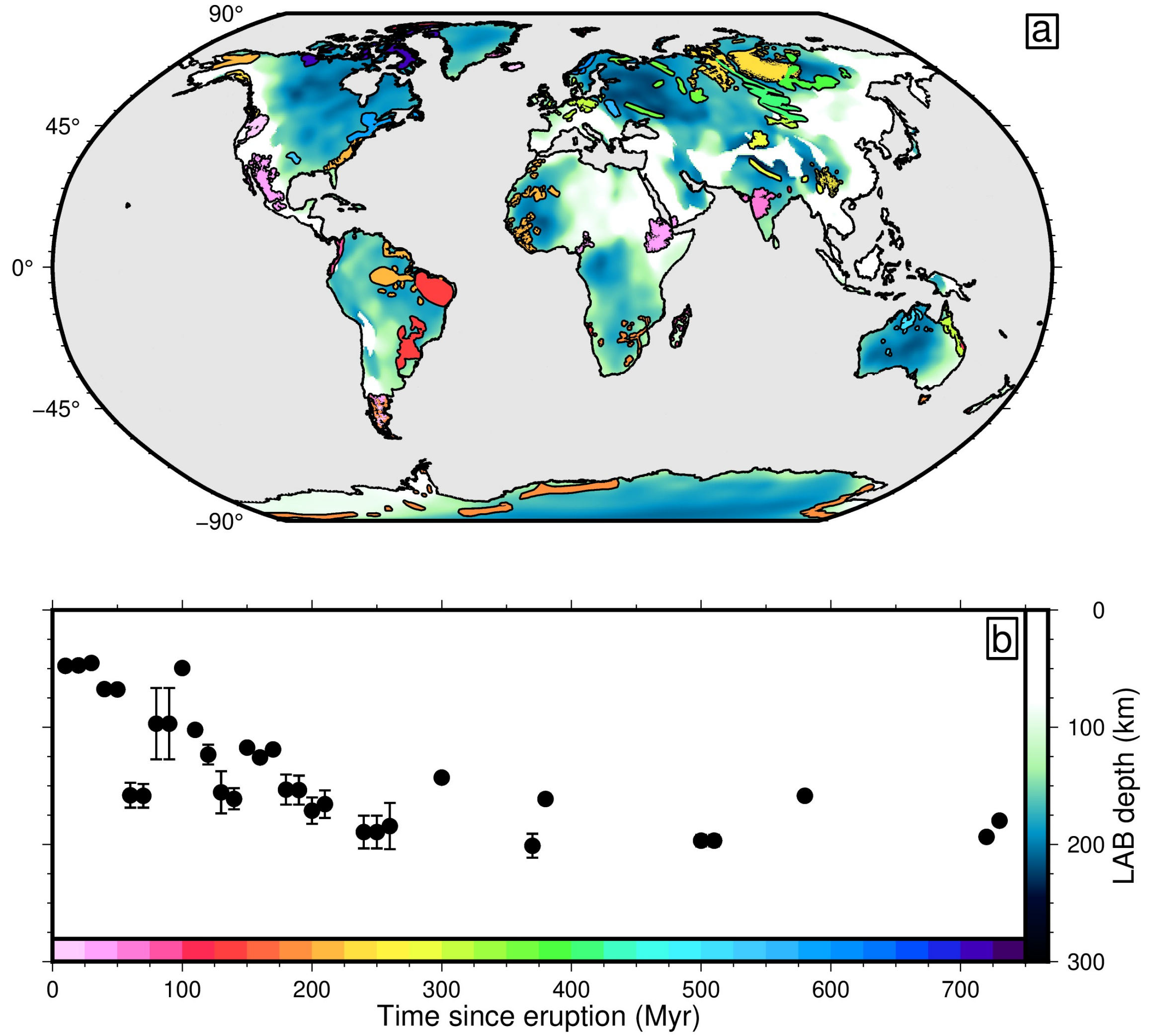


Figure S9: **Global distribution of continental large igneous provinces exploiting** CAM2016 **tomographic model (*115*).** (a) CAM2016 model converted to lithospheric thickness (*8*). Polygons = distribution of continental large igneous provinces in space and time. Scale bars located on axes of panel (b). (b) Modern-day lithospheric thickness beneath large igneous provinces as a function of LIP age.

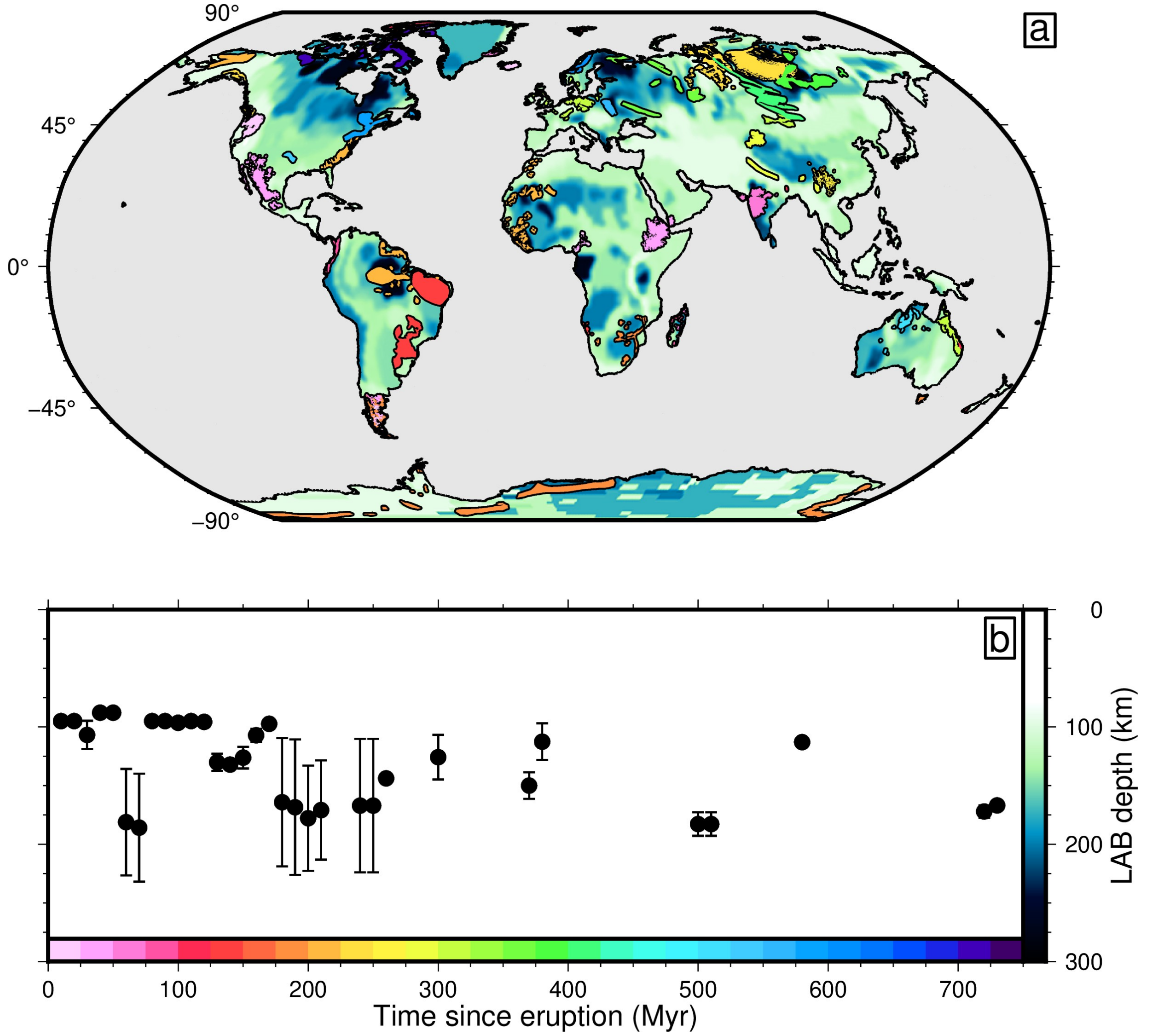


Figure S10: **Global distribution of continental large igneous provinces exploiting** A2006 **model based on heatflow measurements (*114*).** (a) A2006 model. Polygons = distribution of continental large igneous provinces in space and time. Scale bars located on axes of panel (b). (b) Modern-day lithospheric thickness beneath large igneous provinces as a function of LIP age.

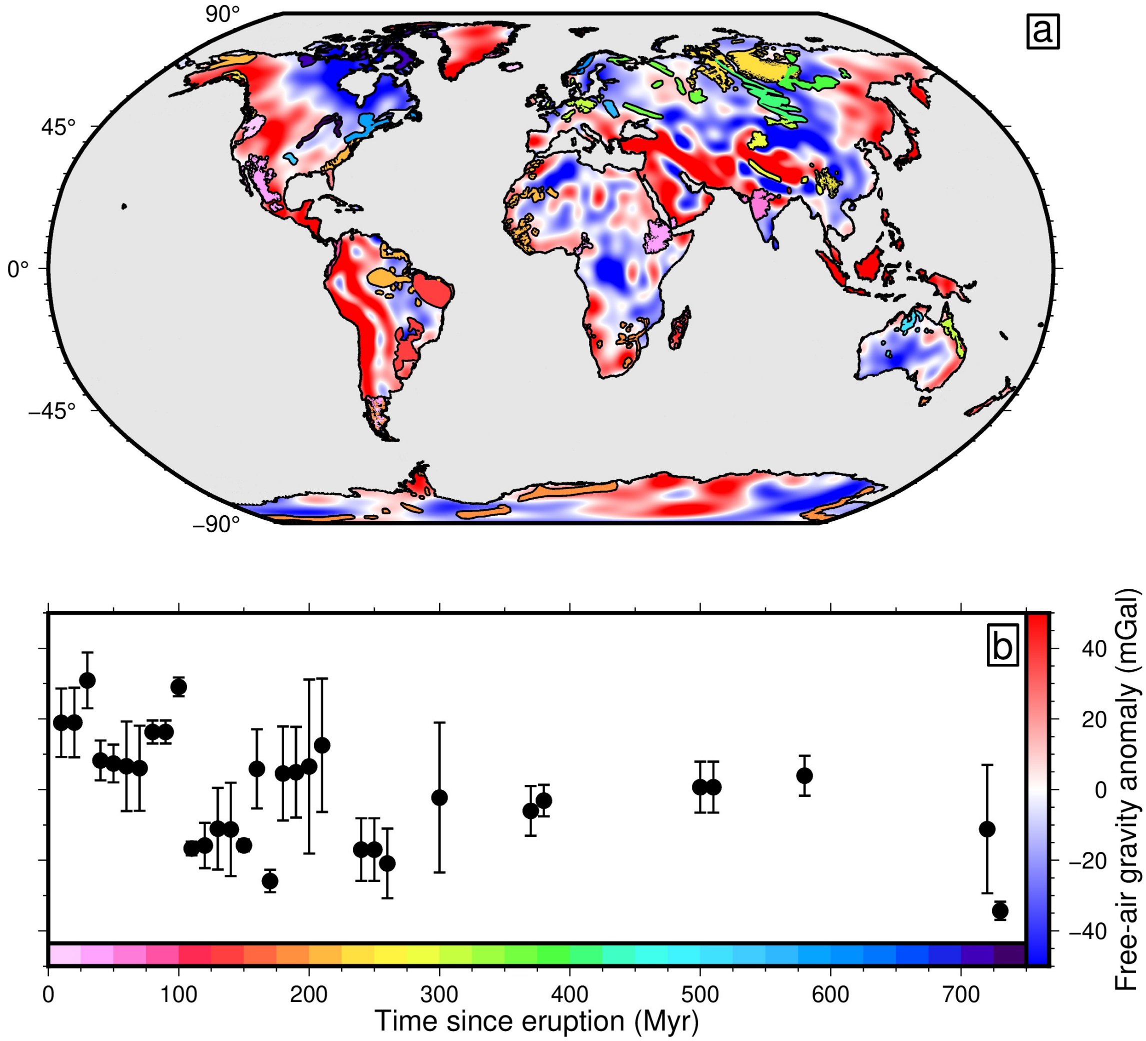


Figure S11: **Global distribution of continental large igneous provinces exploiting** GOCO-03 **gravity model.** (a) Distribution of continental large igneous provinces in space and time. Scale bars located on axes of panel (b). (b) Long-wavelength free-air gravity anomaly beneath large igneous provinces as a function of LIP age.

### 4.3 Alternative LIP-LAB relationships

Since lava flows and dykes can propagate long distances from eruption centres, we explore the relationship between LAB depth beneath large igneous provinces and the time since their eruption using two alternative databases of LIP locations. First, we used the database presented by *Torsvik et al.* (*40*), who mapped the locations of plume centres, accounting for the lateral transport of magmatic materials in lava flows and dykes. They provided information about LIPs extending only back to the Siberian Traps at the and of the Permian Period (i.e. $\sim$ 250 Ma). Figure S12a shows the result of repeating our analysis using the database of *Torsvik et al.* (*40*). A clear positive correlation exists between LAB depth and time since eruption, corroborating our main conclusions. Figures S12c–f show this relationship repeated for various alternative lithospheric thickness models, included those based upon seismic shear-wave tomography and the A2006 model, which is derived from spot-measurements of heatflow (*114*). In all cases a positive relationship exists between LAB depth and time since eruption. Although the relationship is less clear for the A2006 model, older LIPs are still preferentially found on thicker lithosphere, while younger ones never overlie thick lithosphere. Finally, we also carry out our analysis using the long-wavelength frtee-air gravity anomaly, which has been filtered for wavelengths longer than 700 km. This gravity anomaly provides a crude proxy for regions of thick, cold lithospheric mantle and should correlate to a certain extent with LAB depth. Note that we multiply the gravity anomaly in the oceans by $2/5$ to account for differences between water and air-loading. The negative relationship between the free-air gravity anomaly and time since eruption shown on Figure S12g is therefore consistent with our previous results.

Secondly, we compile a new, independent database of plume centres for continental LIPs based upon more recent analyses [Database S5; refs (*43, 52, 55, 120–130*)]. This database includes mostly location data compiled since the publication of *Torsvik et al.* (*40*), including events occurring prior to the Siberian Traps. We exclude locations where we were unable to infer the location of the plume centre from the literature. Figure S13 shows the depth to the LAB beneath each LIP eruption centre as a function of the time since eruption. We find that there is a positive correlation between tomographically determined LAB depth estimates and time since eruption using the SL2013sv, CAM2016, SLNAAFSA, 3D2015-075Sv. Again a clear negative relationship exists between the long-wavelength free-air gravity anomaly and the time since eruption. The relationship between the time of emplacement and the A2006 model is less clear.

## 5 Thermal modelling

### 5.1 Deccan Traps and modelling results

In the main text, we exclude the Deccan traps from our analysis because it is a clear outlier. Its eruption into a narrow rift may mean that the thin spot underlying it is smaller than can be resolved in a global tomographic model. Furthermore, regional and local seismic studies studies have suggested that global tomographic models may overestimate the lithospheric thickness in this region [e.g. (*131*)]. Furthermore, magnetotelluric data suggest the presence of a high-conductivity layer at depths of 80–120 km (*132*). The authors suggest that this layer could be a mid-lithospheric conductive layer but the possibility that it represents the LAB remains. Finally, heatflow data are somewhat ambiguous, with the Deccan traps region yielding lithospheric thickness values ranging between 50–200 km (*114, 133*). Furthermore, these two studies define the LAB as the intersection of a calculated lithospheric geotherm with the 1330 °C mantle adiabat, and the mantle solidus, respectively. Hence there are systematic differences between LAB depth in these studies compared to this study. We repeat our modelling here, this time including the Deccan traps. We find that the effect on our results are minimal (Figure S14). $z^{\infty}_{LAB}$ remains constant at 195 km, while $z_p$ increases from 248 to 249 km and $(T_p)_0$ decreases from 1465°C to 1413°C. The minimum misfit value also increases slightly.

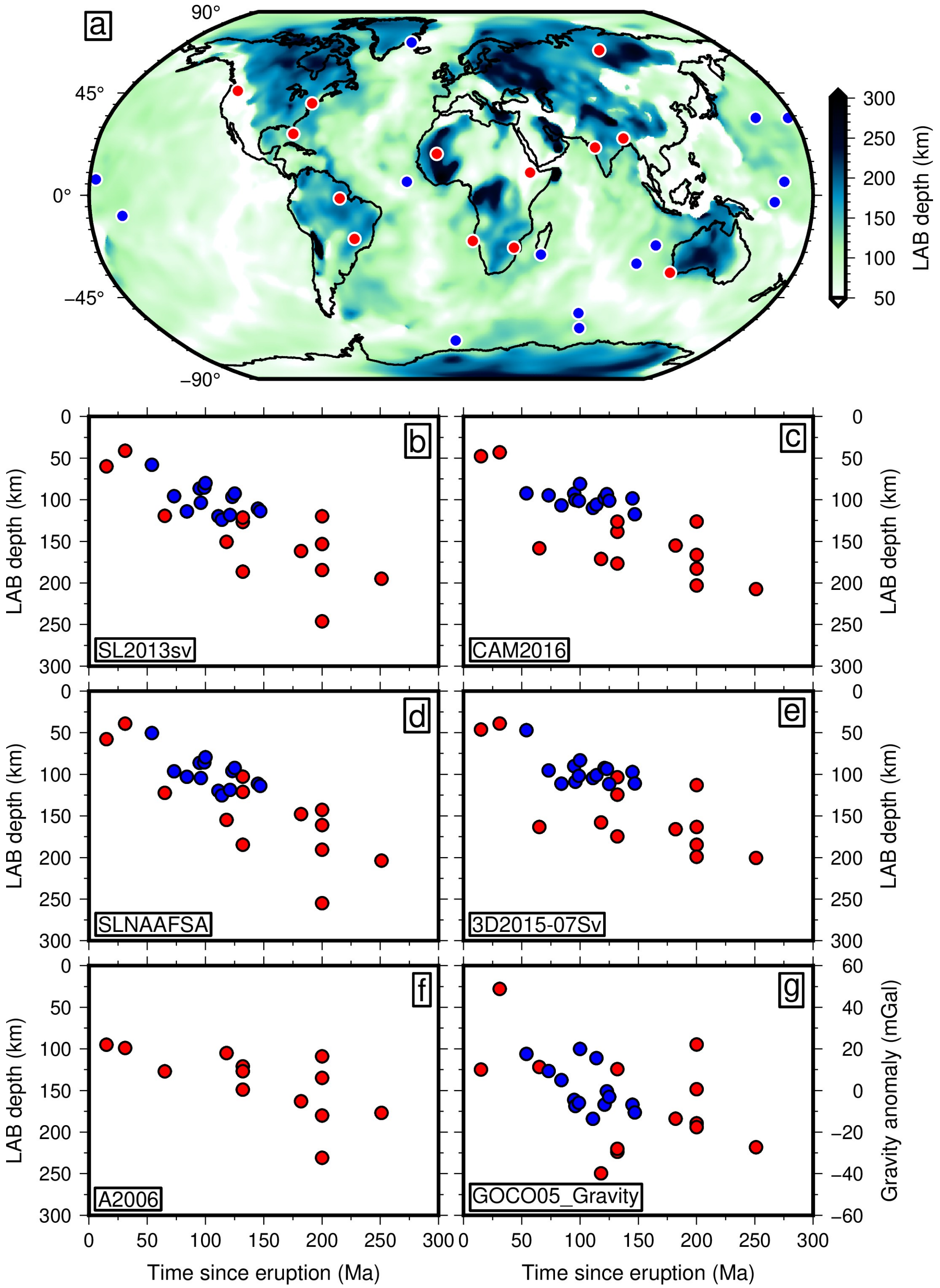


Figure S12: ***Torsvik et al.* (*40*) large igneous province database**. (a) Distribution of large igneous provinces mapped by *Torsvik et al.* (*40*) on lithospheric thickness model of *Hoggard et al.* (*8*). Blue circles = oceanic LIPs; red circles = continental LIPs. (b) LAB depth as a function of time since eruption for SL2013sv model (*8, 75*). Symbols same as panel (a). (c) CAM2016 model (*8, 119*). (d) SLNAAFSA merged model (*8, 19, 116, 117*). (e) 3D2015-07Sv (*8, 118*). (f) A2006 (*114*). (g) GOCO05 free-air gravity model filtered for wavelengths 800 < $\delta g$ < 2500 km.

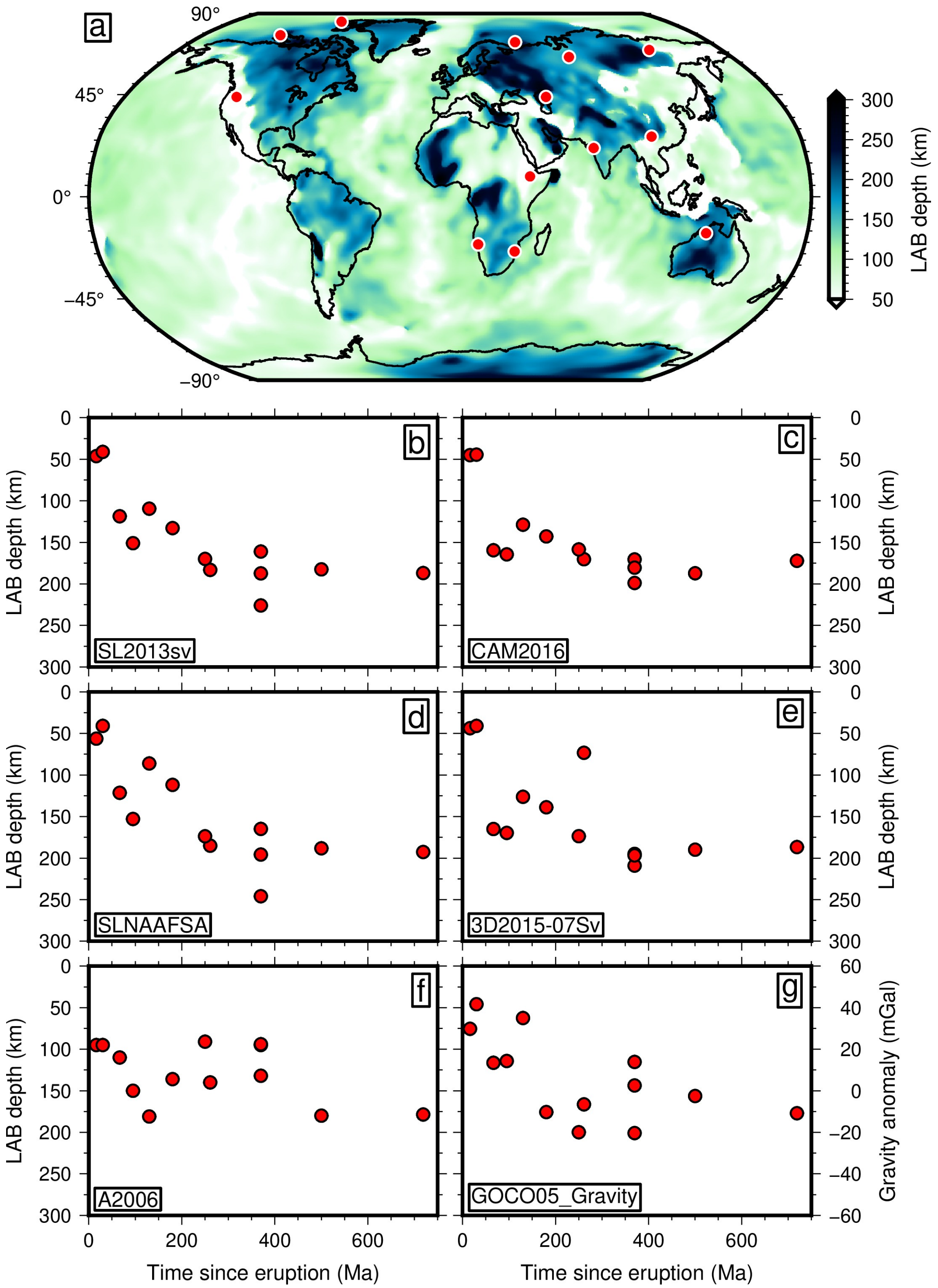


Figure S13: **Database of LIP eruption loci (this study)**. (a) Distribution of LIP eruption loci compiled from literature (see Table 1) on lithospheric thickness model of *Hoggard et al.* (*8*). Blue circles = oceanic LIPs; red circles = continental LIPs. (b) LAB depth as a function of time since eruption for SL2013sv model (*8, 75*). Symbols same as panel (a). (c) CAM2016 model (*8, 119*). (d) SLNAAFSA merged model (*8, 19, 116, 117*). (e) 3D2015-07Sv (*8, 118*). (f) A2006 (*114*). (g) GOCO05 free-air gravity model filtered for wavelengths 800 < $\delta g$ < 2500 km.

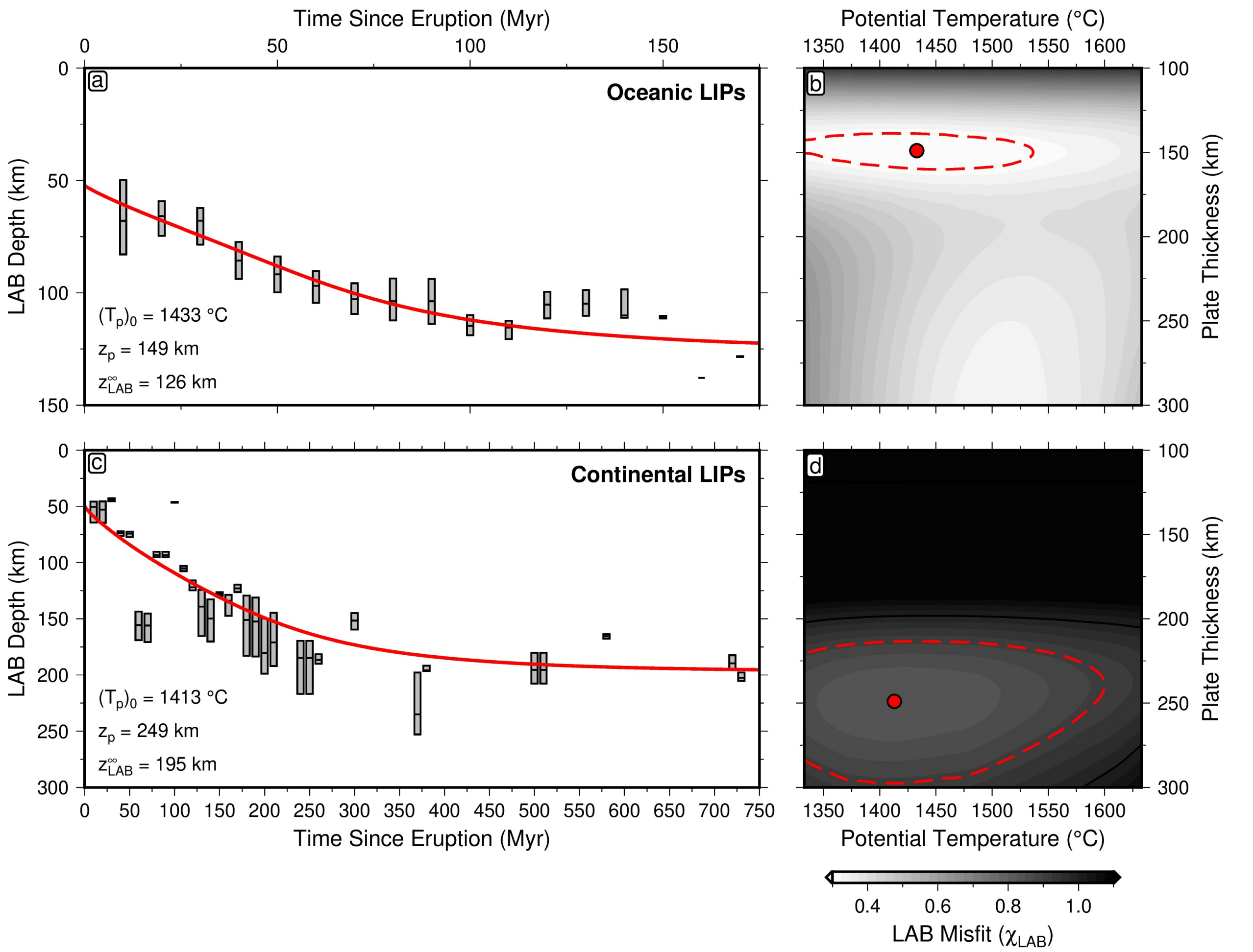


Figure S14: **Lithospheric thermal modelling including Deccan Traps.** (a) Observed and modelled LAB depth beneath oceanic intraplate magmatic provinces as function of eruptive age. Grey bar = interquartile range of lithospheric thickness distribution. Red line = best-fitting modelled LAB depth. $(T_p)_0$ = initial potential temperature; $z_p$ = plate thickness; $z^{\infty}_{LAB}$ = asymptotic depth to the LAB as $t \rightarrow \infty$. (b) Misfit between observed and calculated lithosphere-asthenosphere boundary (LAB) depth as function of plate thickness, $z_p$, and potential temperature, $T_p$ for oceanic intraplate magmatism (see methods for details). Red circle = misfit minimum; red dashed line = contour marking 1.5× value at misfit minimum. Optimum values given in lower left-hand corner of panel (a). (c) Observed and modelled LAB depth beneath Continental LIPs as a function eruptive age. Red bars = Deccan Traps, excluded from the analysis. (d) As panel (b) but for continental LIPs. Optimum values given in lower left-hand corner of panel (c).

## 5.2 Alternative model boundary conditions

Here we test the effects of two conditions on our modelling. First, we assess the impact of using a steady-state starting geothermal gradient within the lithosphere at the emplacement time of the LIP. Secondly we explore the impact of changing our paramterisation of the initial potential temperature (i.e. $(T_P)_0$ at the lower boundary.

### 5.2.1 Discontinuous starting geotherm

In the main text, we present thermal modelling that assumes that the initial geothermal gradient at $t = 0$ Ma is in steady-state (i.e. the lithosphere is thermally equilibrated). It is possible, however that the initial geothermal gradients is not equilibrated at $t = 0$ Ma. If lithospheric mantle is instantaneously removed from the base of the plate from a thickness of $z^{\infty}_{LAB}$ to $z^{0}_{LAB}$, then the geotherm within the lithosphere will match that of $z^{\infty}_{LAB}$, before encountering a discontinuity at depth $z^{0}_{LAB}$ at which it jumps onto the mantle adiabat that corresponds to $(T_p)_0$ [e.g. (*51, 78*)].

Figure S15 shows the effect for both oceanic and continental igneous provinces of using a discontinuous starting geotherm. All other model parameters remain the same as those described for our optimal model (i.e. with continuous initial geothermal gradient) in the main text. In general, since the lithosphere is initially cooler, the LAB deepens more rapidly than in the equilibrated case, however $\frac{\mathrm{d}z_{LAB}}{\mathrm{d}t}$ rapidly approaches that in the equilibrated case. Nonetheless, using a discontinuous initial temperature profile is likely to have a small effect on our optimal values of $z_p$ and $(T_p)_0$ since the LAB is about $\sim$ 10 km deeper for the first 60–70Ma than for the equilibrated initial condition. To fit observed LAB depths, it is likely that a slightly larger asthenospheric thermal anomaly would be needed to mitigate this rapid initial deepening of the LAB. These two models represent end-member starting conditions with the true starting geotherm likely to lie somewhere in between. However, if LIPs are emplaced over 5–10 Ma, then we note that adequate time may have elapsed to to approach thermal equilibrium prior to re-thickening if the plate was rapidly mechanically thinned prior to magmatism.

### 5.2.2 Evolving basal temperature condition

In the main text, we impose an evolving basal boundary condition on our plate model in order to simulate the effect of a mantle plume. Here, we explore first the effect of varying the decay time. We vary the time taken for $T_p$ to decrease linearly from from $(T_p)_0$ to $T_p = 1333$ °C from 10–100 Ma. Secondly, we alter the parametrisation so that instead the temperature anomaly decays exponentially from $(T_p)_0$ to $T_p = 1333$ °C with an e-folding time of 3.33-33.3 Ma. Finally, we vary the plume sinking rate, $v_{plume}$, from 1-15 mm yr$^{-1}$. We find that varying these parameters has a minimal impact on our results (i.e. maximum change in $z_{LAB}$ of 7%, which is significantly below the error in our observed values of $z^{1}_{LAB}$ (Figure S16). It is important to note, however, that imposing an excess temperature anomaly, regardless of its exact parametrisation, greatly improves the fit to the data.

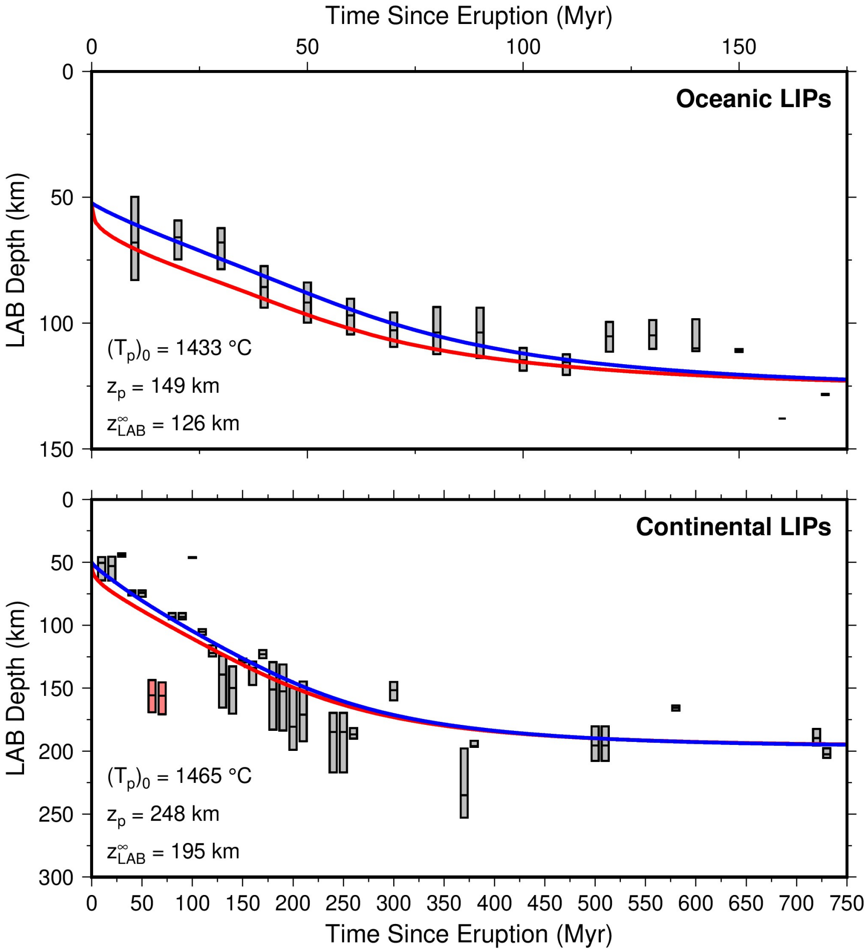


Figure S15: **Effect of including discontinuous initial geothermal gradient.** (a) Observed and modelled LAB depth beneath oceanic intraplate magmatic provinces as function of eruptive age. Grey bar = interquartile range of lithospheric thickness distribution. Blue line = best-fitting modelled LAB depth for continuous initial geotherm. Red line = LAB depth as a function of time for discontinuous initial geotherm (i.e. with step at base of lithosphere), all other parameters same as for blue line. $(T_p)_0$ = initial potential temperature; $z_p$ = plate thickness; $z^{\infty}_{LAB}$ = asymptotic depth to the LAB as $t \longrightarrow \infty$; all values calculated using continuous starting geotherm (i.e. same as Figure 3 of main text). (b) Same as panel (a) but for continental LIPs. Red bars = Deccan traps, excluded from analysis.

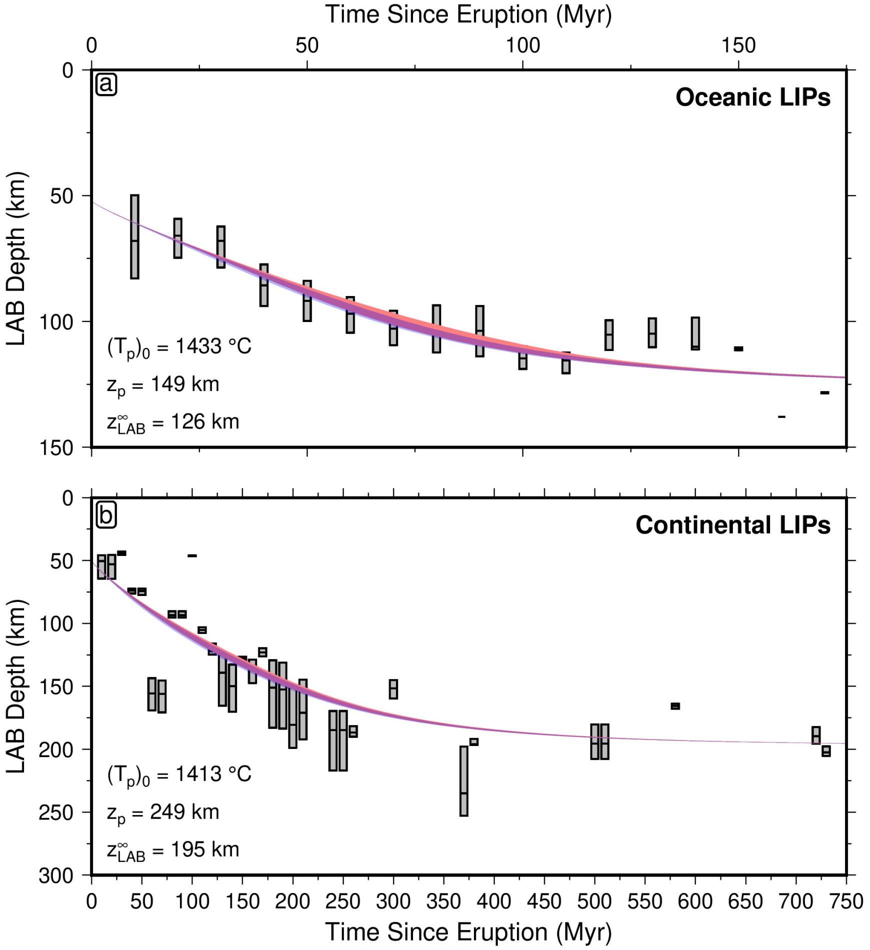


Figure S16: **Effect of changing parameterisation of decay in basal potential temperature.** (a) Observed and modelled LAB depth beneath oceanic intraplate magmatic provinces as function of time since eruption. Grey bar = interquartile range of lithospheric thickness distribution. Red envelope = range of predicted values of $z_{LAB}(t)$ with linear decay from $(T_P)_0$ to 1333°C over 10-100 Ma. Blue envelope = range of predicted values using exponential decay function where e-folding time is 1/3 of the decay times for the linear case.

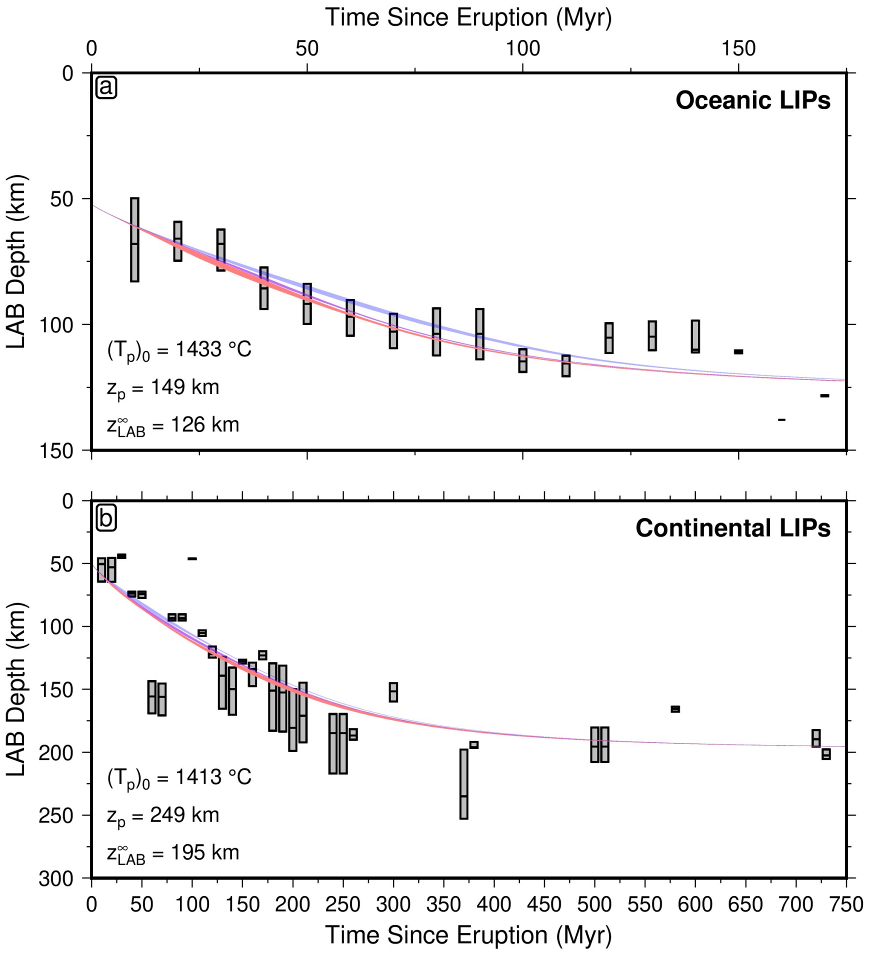


Figure S17: **Effect of changing sinking rate of boundary between plume head and base of thermal boundary layer.** (a) Observed and modelled LAB depth beneath oceanic intraplate magmatic provinces as function of time since eruption. Grey bar = interquartile range of lithospheric thickness distribution. Red envelope = range of predicted values of $z_{LAB}(t)$ with linear decay from $(T_P)_0$ to 1333°C over 10-100 Ma. Red envelope = range of predicted values by varying plume sinking rate from 1-15 mm yr$^{-1}$ when linear temperature decay lasts 10 Ma. Purple envelope = range of predicted values when linear decay lasts 30 Ma. Blue envelope = range when linear decay lasts 100 Ma.

## REFERENCES AND NOTES

1. D. McKenzie, M. J. Bickle, The volume and composition of melt generated by extension of the lithosphere. *J. Petrol.* **29**, 625–679 (1988).

2. S. D. King, D. L. Anderson, Edge-driven convection. *Earth Planet. Sci. Lett.* **160**, 289–296 (1998).

3. N. H. Sleep, Survival of Archean cratonal lithosphere. *J. Geophys. Res. Solid Earth* **108** (2003).

4. A. Lenardic, L.-N. Moresi, H. Mühlhaus, Longevity and stability of cratonic lithosphere: Insights from numerical simulations of coupled mantle convection and continental tectonics. *J. Geophys. Res. Solid Earth* **108** (2003).

5. D. R. Davies, N. Rawlinson, G. Iaffaldano, I. H. Campbell, Lithospheric controls on magma composition along Earth's longest continental hotspot track. *Nature* **525**, 511–514 (2015).

6. Y. Niu, Lithosphere thickness controls the extent of mantle melting, depth of melt extraction and basalt compositions in all tectonic settings on earth—A review and new perspectives. *Earth Sci. Rev.* **217**, 103614 (2021).

7. P. Molnar, P. England, Late Cenozoic uplift of mountain ranges and global climate change: Chicken or egg? *Nature* **346**, 29–34 (1990).

8. M. J. Hoggard, K. Czarnota, F. D. Richards, D. L. Huston, A. L. Jaques, S. Ghelichkhan , Global distribution of sediment-hosted metals controlled by craton edge stability. *Nat. Geosci.* **13**, 504–510 (2020).

9. M. Hoggard, J. Austermann, C. Randel, S. Stephenson, Observational estimates of dynamic topography through space and time, in *Mantle Convection and Surface Expressions* (American Geophysical Union, 2021), pp. 371–411.

10. B. Parsons, J. G. Sclater, An analysis of the variation of ocean floor bathymetry and heat flow with age. *J. Geophys. Res.* **82**, 803–827 (1977).

11. J. Jackson, D. McKenzie, K. Priestley, B. Emmerson, New views on the structure and rheology of the lithosphere. *J. Geol. Soc.* **165**, 453–465 (2008).

12. W. L. Griffin, S. Y. O'Reilly, J. C. Afonso, G. C. Begg, The composition and evolution of lithospheric mantle: A re-evaluation and its tectonic implications. *J. Petrol.* **50**, 1185–1204 (2009).

13. M. E. Pasyanos, T. G. Masters, G. Laske, Z. Ma, LITHO1.0: An updated crust and lithospheric model of the Earth. *J. Geophys. Res. Solid Earth* **119**, 2153–2173 (2014).

14. J. C. Afonso, N. Rawlinson, Y. Yang, D. L. Schutt, A. G. Jones, J. Fullea, W. L. Griffin, 3-D multiobservable probabilistic inversion for the compositional and thermal structure of the lithosphere

and upper mantle: III. Thermochemical tomography in the Western-Central U.S. *J. Geophys. Res. Solid Earth* **121**, 7337–7370 (2016).

15. D. G. Pearson, J. M. Scott, J. Liu, A. Schaeffer, L. H. Wang, J. van Hunen, K. Szilas, T. Chacko, P. B. Kelemen , Deep continental roots and cratons. *Nature* **596**, 199–210 (2021).
16. T. H. Jordan, Composition and development of the continental tectosphere. *Nature* **274**, 544–548 (1978).
17. C. J. O'Neill, A. Lenardic, W. L. Griffin, S. Y. O'Reilly, Dynamics of cratons in an evolving mantle. *Lithos* **102**, 12–24 (2008).
18. R. E. Ernst, A. Dickson, A. Bekker, in *Large Igneous Provinces: A Driver of Global Environmental and Biotic Changes* , R. E. Ernst, A. J. Dickson, A. Becker, eds. (American Geophysical Union, 2021), vol. 255 of *Geophysical Monograph Series*, pp. 3–26.
19. N. L. Celli, S. Lebedev, A. J. Schaeffer, M. Ravenna, C. Gaina, The upper mantle beneath the South Atlantic Ocean, South America and Africa from waveform tomography with massive data sets. *Geophys. J. Int.* **221**, 178–204 (2020).
20. P. W. Ball, N. J. White, J. Maclennan, S. N. Stephenson, Global influence of mantle temperature and plate thickness on intraplate volcanism. *Nat. Commun.* **12**, 2045 (2021).
21. J. Liu, D. G. Pearson, L. H. Wang, K. A. Mather, B. A. Kjarsgaard, A. J. Schaeffer, G. J. Irvine, M. G. Kopylova, J. P. Armstrong, Plume-driven recratonization of deep continental lithospheric mantle. *Nature* **592**, 732–736 (2021).
22. C. J. Grose, J. C. Afonso, Comprehensive plate models for the thermal evolution of oceanic lithosphere. *Geochem. Geophys. Geosyst.* **14**, 3751–3778 (2013).
23. F. D. Richards, M. J. Hoggard, L. Cowton, N. J. White, Reassessing the Thermal Structure of Oceanic Lithosphere With Revised Global Inventories of Basement Depths and Heat Flow Measurements. *J. Geophys. Res. Solid Earth* **123**, 9136–9161 (2018).
24. F. D. Richards, M. J. Hoggard, N. White, S. Ghelichkhan, Quantifying the relationship between short-wavelength dynamic topography and thermomechanical structure of the upper mantle using calibrated parameterization of anelasticity. *J. Geophys. Res. Solid Earth* e2019JB019062 (2020).
25. T. Plank, D. Forsyth, Thermal structure and melting conditions in the mantle beneath the Basin and Range province from seismology and petrology. *Geochem. Geophys. Geosyst.* **17**, 1312–1338 (2016).

26. F. McNab, P. W. Ball, meltPT: A Python package for basaltic whole-rock thermobarometric analysis with application to Hawai'i. *Volcanica* **6**, 63–76 (2023).
27. R. D. Müller, N. Flament, K. J. Matthews, S. E. Williams, M. Gurnis, Formation of Australian continental margin highlands driven by plate–mantle interaction. *Earth Planet. Sci. Lett.* **441**, 60–70 (2016).
28. F. Richards, M. Hoggard, A. Crosby, S. Ghelichkhan, N. J. White, Structure and dynamics of the oceanic lithosphere-asthenosphere system. *Phys. Earth Planet. In.* **309**, 106559 (2020b).
29. F. A. Darbyshire, I. T. Bjarnason, R. S. White, Ó. G. Flóvenz, Crustal structure above the iceland mantle plume imaged by the icemelt refraction profile. *Geophys. J. Int.* **135**, 1131–1149 (1998).
30. J. Jenkins, J. Maclennan, R. G. Green, S. Cottaar, A. F. Deuss, R. S. White, Crustal formation on a spreading ridge above a mantle plume: Receiver function imaging of the icelandic crust. *J. Geophys. Res. Solid Earth* **123**, 5190–5208 (2018).
31. B. M. Le, T. Yang, J. P. Morgan, Seismic constraints on crustal and uppermost mantle structure beneath the hawaiian swell: Implications for plume-lithosphere interactions. *J. Geophys. Res. Solid Earth* **127**, *e2021JB023822* (2022).
32. N. Schmerr, The gutenberg discontinuity: Melt at the lithosphere-asthenosphere boundary. *Science* **335**, 1480–1483 (2012).
33. I. Guest, G. Ito, M. O. Garcia, E. Hellebrand, Extensive magmatic heating of the lithosphere beneath the Hawaiian Islands inferred from salt lake crater mantle xenoliths. *Geochem. Geophys. Geosyst.* **21**, e2020GC009359 (2020).
34. A. Pleus, G. Ito, P. Wessel, L. N. Frazer, Rheology and thermal structure of the lithosphere beneath the Hawaiian ridge inferred from gravity data and models of plate flexure. *Geophys. J. Int.* **222**, 207–224 (2020).
35. M. F. Coffin, O. Eldholm, Large igneous provinces: Crustal structure, dimensions, and external consequences. *Rev. Geophys.* **32**, 1 (1994).
36. M. F. Coffin, R. A. Duncan, O. Eldholm, J. G. Fitton, F. A. Frey, H. C. Larsen, J. J. Mahoney, A. D. Saunders, R. Schlich, P. J. Wallace, Large igneous provinces and scientific ocean drilling: Status quo and a look ahead. *Oceanography* **19**, 150–160 (2006).
37. R. S. Detrick, S. T. Crough, Island subsidence, hot spots, and lithospheric thinning. *J. Geophys. Res. Solid Earth* **83**, 1236 (1978).

38. K. L. Huppert, J. T. Perron, L. H. Royden, Hotspot swells and the lifespan of volcanic ocean islands. *Sci. Adv.* **6**, eaaw6906 (2020).
39. R. S. White, D. McKenzie, Mantle plumes and flood basalts. *J. Geophys. Res. Solid Earth* **100**, 17543–17585 (1995).
40. T. H. Torsvik, M. A. Smethurst, K. Burke, B. Steinberger, Large igneous provinces generated from the margins of the large low-velocity provinces in the deep mantle. *Geophys. J. Int.* **167**, 1447–1460 (2006).
41. C. A. Stein, S. Stein, Constraints on hydrothermal heat flux through the oceanic lithosphere from global heat flow. *J. Geophys. Res. Solid Earth* **99**, 3081–3095 (1994).
42. A. G. Whittington, A. M. Hofmeister, P. I. Nabelek, Temperature-dependent thermal diffusivity of the earth's crust and implications for magmatism. *Nature* **458**, 319–321 (2009).
43. A. D. Saunders, S. M. Jones, L. A. Morgan, K. L. Pierce, M. Widdowson, Y. G. Xu, Regional uplift associated with continental large igneous provinces: The roles of mantle plumes and the lithosphere. *Chem. Geol.* **241**, 282–318 (2007).
44. R. Newman, N. White, The dynamics of extensional sedimentary basins: Constraints from subsidence inversion. *Philos. Trans. R. Soc. A* **357**, 805–834 (1999).
45. J. J. Armitage, P. A. Allen, Cratonic basins and the long-term subsidence history of continental interiors. *J. Geol. Soc.* **167**, 61–70 (2010).
46. C.-T. A. Lee, P. Luffi, E. J. Chin, Building and destroying continental mantle. *Annu. Rev. Earth Planet. Sci.* **39**, 59–90 (2011).
47. P. Bird, Continental delamination and the colorado plateau. *J. Geophys. Res. Solid Earth* **84**, 7561–7571 (1979).
48. D. McKenzie, Some remarks on the development of sedimentary basins. *Earth Planet. Sci. Lett.* **40**, 25–32 (1978).
49. B. He, Y.-G. Xu, S.-L. Chung, L. Xiao, Y. Wang, Sedimentary evidence for a rapid, kilometer-scale crustal doming prior to the eruption of the emeishan flood basalts. *Earth Planet. Sci. Lett.* **213**, 391–405 (2003).
50. X. Wang, L. Shao, K. A. Eriksson, Z. Yan, J. Wang, H. Li, R. Zhou, J. Lu, Evolution of a plume-influenced source-to-sink system: An example from the coupled central emeishan large igneous province and adjacent western yangtze cratonic basin in the late permian, sw china. *Earth Sci. Rev.* **207**, 103224 (2020).

51. S. N. Stephenson, N. J. White, A. Carter, D. Seward, P. W. Ball, M. Klöcking, Cenozoic dynamic topography of Madagascar. *Geochem. Geophys. Geosyst.* **22**, e2020GC009624 (2021).
52. A. Sembroni, C. Faccenna, T. W. Becker, P. Molin, B. Abebe, Long-term, deep-mantle support of the Ethiopia-Yemen plateau. *Tectonics* **35**, 469–488 (2016).
53. M. Klöcking, M. J. Hoggard, V. Rodríguez Tribaldos, F. D. Richards, A. R. Guimarães, J. Maclennan, N. J. White , A tale of two domes: Neogene to recent volcanism and dynamic uplift of northeast Brazil and southwest Africa. *Earth Planet. Sci. Lett.* **547**, 116464 (2020).
54. J. Brodie, N. White, Sedimentary basin inversion caused by igneous underplating: Northwest european continental shelf. *Geology* **22**, 147–150 (1994).
55. K. L. Buchan, R. E. Ernst, A giant circumferential dyke swarm associated with the high arctic large igneous province (halip). *Gondw. Res.* **58**, 39–57 (2018).
56. J. De Laat, S. Lebedev, N. L. Celli, R. Bonadio, B. Chagas de Melo, N. Rawlinson, Structure and evolution of the Australian plate and underlying upper mantle from waveform tomography with massive data sets. *Geophys. J. Int.* **234**, 153–189 (2023).
57. A. Boscaini, A. Marzoli, H. Bertrand, M. Chiaradia, F. Jourdan, M. Faccenda, C. M. Meyzen, S. Callegaro, L. Serrano Durán , Cratonic keels controlled the emplacement of the central atlantic magmatic province (camp). *Earth Planet. Sci. Lett.* **584**, 117480 (2022).
58. K. A. Mather, D. G. Pearson, D. McKenzie, B. A. Kjarsgaard, K. Priestley, Constraints on the depth and thermal history of cratonic lithosphere from peridotite xenoliths, xenocrysts and seismology. *Lithos* **125**, 729–742 (2011).
59. S. F. Foley, Rejuvenation and erosion of the cratonic lithosphere. *Nat. Geosci.* **1**, 503–510 (2008).
60. J. Hu, L. Liu, M. Faccenda, Q. Zhou, K. M. Fischer, S. Marshak, C. Lundstrom, Modification of the western gondwana craton by plume–lithosphere interaction. *Nat. Geosci.* **11**, 203–210 (2018).
61. D. McKenzie, F. M. Richter, Parameterized thermal convection in a layered region and the thermal history of the earth. *J. Geophys. Res. Solid Earth* **86**, 11667 (1981).
62. A. H. Peslier, A. B. Woodland, D. R. Bell, M. Lazarov, Olivine water contents in the continental lithosphere and the longevity of cratons. *Nature* **467**, 78–81 (2010).
63. T. Stachel, R. W. Luth, Diamond formation—Where, when and how? *Lithos* **220-223**, 200–220 (2015).
64. J. Sun, C. Z. Liu, S. Tappe, S. I. Kostrovitsky, F. Y. Wu, D. Yakovlev, Y. H. Yang, J. H. Yang, Repeated kimberlite magmatism beneath Yakutia and its relationship to Siberian flood volcanism:

Insights from in situ U–Pb and Sr–Nd perovskite isotope analysis. *Earth Planet. Sci. Lett.* **404**, 283–295 (2014).

65. K. V. Smit, T. Stachel, R. A. Stern, Diamonds in the Attawapiskat area of the Superior craton (Canada): Evidence for a major diamond-forming event younger than 1.1 Ga. *Contrib. Mineral. Petrol.* **167**, 962 (2014).
66. H. Helmstaedt, J. Gurney, Geotectonic controls of primary diamond deposits: Implications for area selection. *J. Geochem. Explor.* **53**, 125–144 (1995).
67. R. E. Ernst, D. R. Davies, S. M. Jowitt, I. H. Campbell, When do mantle plumes destroy diamonds? *Earth Planet. Sci. Lett.* **502**, 244–252 (2018).
68. H. Sigurdsson, Evidence of volcanic loading of the atmosphere and climate response. *Global Planet. Change* **3**, 277–289 (1990).
69. B. A. Black, L. T. Elkins-Tanton, M. C. Rowe, I. U. Peate, Magnitude and consequences of volatile release from the Siberian Traps. *Earth Planet. Sci. Lett.* **317**, 363 (2012).
70. S. M. Stanley, Estimates of the magnitudes of major marine mass extinctions in earth history. *Proc. Natl. Acad. Sci. U.S.A.* **113**, E6325–E6334 (2016).
71. M. W. Broadley, P. H. Barry, C. J. Ballentine, L. A. Taylor, R. Burgess, End-Permian extinction amplified by plume-induced release of recycled lithospheric volatiles. *Nat. Geosci.* **11**, 682–687 (2018).
72. C. Ganino, N. T. Arndt, Climate changes caused by degassing of sediments during the emplacement of large igneous provinces. *Geology* **37**, 323–326 (2009).
73. J. Guex, S. Pilet, O. Müntener, A. Bartolini, J. Spangenberg, B. Schoene, B. Sell, U. Schaltegger, Thermal erosion of cratonic lithosphere as a potential trigger for mass-extinction. *Sci. Rep.* **6**, 23168 (2016).
74. M.-F. Zhou, J. Malpas, X. Y. Song, P. T. Robinson, M. Sun, A. K. Kennedy, C. M. Lesher, R. R. Keays, A temporal link between the Emeishan large igneous province (SW China) and the end-Guadalupian mass extinction. *Earth Planet. Sci. Lett.* **196**, 113–122 (2002).
75. A. J. Schaeffer, S. Lebedev, Global shear speed structure of the upper mantle and transition zone. *Geophys. J. Int.* **194**, 417–449 (2013).
76. H. Yamauchi, Y. Takei, Polycrystal anelasticity at near-solidus temperatures. *J. Geophys. Res. Solid Earth* **121**, 7790–7820 (2016).

77. J. E. Dixon, L. Leist, C. Langmuir, J.-G. Schilling, Recycled dehydrated lithosphere observed in plume-influenced mid-ocean-ridge basalt. *Nature* **420**, 385–389 (2002).
78. F. McNab, P. W. Ball, M. J. Hoggard, N. J. White, Neogene uplift and magmatism of Anatolia: Insights from drainage analysis and basaltic geochemistry. *Geochem. Geophys. Geosyst.* **19**, 175–213 (2018).
79. C. T. A. Lee, P. Luffi, T. Plank, H. Dalton, W. P. Leeman, Constraints on the depths and temperatures of basaltic magma generation on Earth and other terrestrial planets using new thermobarometers for mafic magmas. *Earth Planet. Sci. Lett.* **279**, 20–33 (2009).
80. D. Canil, R. D. Hyndman, D. Fode, Hygrometric control on the lithosphere-asthenosphere boundary: A 28 million year record from the Canadian Cordillera. *Geophys. Res. Lett.* **48**, e2020GL091957 (2021).
81. D. McKenzie, R. K. O'Nions, Partial melt distributions from inversion of rare earth element concentrations. *J. Petrol.* **32**, 1021–1091 (1991).
82. E. S. Jennings, T. J. B. Holland, A simple thermodynamic model for melting of peridotite in the system NCFMASOCr. *J. Petrol.* **56**, 869–892 (2015).
83. W. H. Press, S. A. Teukolsky, W. T. Vetterling, B. P. Flannery, *Numerical Recipes 3rd Edition: The Art of Scientific Computing* (Cambridge Univ. Press, 2007).
84. D. McKenzie, J. Jackson, K. Priestley, Thermal structure of oceanic and continental lithosphere. *Earth Planet. Sci. Lett.* **233**, 337–349 (2005).
85. M. J. Hoggard, J. Winterbourne, K. Czarnota, N. White, Oceanic residual depth measurements, the plate cooling model and global dynamic topography. *J. Geophys. Res. Solid Earth* **122**, 2328–2372 (2017).
86. J. G. Negi, O. P. Pandey, P. K. Agrawal, Super-mobility of hot indian lithosphere. *Tectonophysics* **131**, 147–156 (1986).
87. J. Sharma, M. R. Kumar, K. S. Roy, P. N. S. Roy, Seismic imprints of plume-lithosphere interaction beneath the Northwestern Deccan Volcanic Province. *J. Geophys. Res. Solid Earth* **123**, 10,831–10,853 (2018).
88. P. K. Patro, S. V. S. Sarma, Lithospheric electrical imaging of the deccan trap covered region of western India. *J. Geophys. Res. Solid Earth* **114** (2009).

89. C. Ebinger, T. Bechtel, D. Forsyth, C. Bowin, Effective elastic plate thickness beneath the East African and Afar plateaus and dynamic compensation of the uplifts. *J. Geophys. Res. Solid Earth* **94**, 2883–2901 (1989).

90. J. Weber, The structures of the Alpha Ridge, Arctic Ocean and Iceland-Faeroe Ridge, North Atlantic: Comparisons and implications for the evolution of the Canada Basin. *Mar. Geol.* **93**, 43–68 (1990).

91. A. Døssing, H. R. Jackson, J. Matzka, I. Einarsson, T. M. Rasmussen, A. V. Olesen, J. M. Brozena, On the origin of the Amerasia Basin and the High Arctic Large Igneous Province—Results of new aeromagnetic data. *Earth Planet. Sci. Lett.* **363**, 219–230 (2013).

92. F. Riefstahl, S. Estrada, W. H. Geissler, W. Jokat, R. Stein, H. Kämpf, P. Dulski, R. Naumann, C. Spiegel , Provenance and characteristics of rocks from the Yermak Plateau, Arctic Ocean: Petrographic, geochemical and geochronological constraints. *Mar. Geol.* **343**, 125–145 (2013).

93. R. A. Duncan, Age progressive volcanism in the new england seamounts and the opening of the central atlantic ocean. *J. Geophys. Res. Solid Earth* **89**, 9980–9990 (1984).

94. R. V. Santos, C. E. Ganade, C. M. Lacasse, I. S. L. Costa, I. Pessanha, E. P. Frazão, E. L. Dantas, J. A. Cavalcante , Dating Gondwanan continental crust at the Rio Grande Rise, South Atlantic. *Terra Nova* **31**, 424–429 (2019).

95. S. Homrighausen, K. Hoernle, F. Hauff, J. A. Wartho, P. van den Bogaard, D. Garbe-Schönberg , New age and geochemical data from the Walvis Ridge: The temporal and spatial diversity of South Atlantic intraplate volcanism and its possible origin. *Geochim. Cosmochim. Acta* **245**, 16–34 (2019).

96. T. M. Maia, A. C. dos Santos, E. R. V. Rocha-Júnior, C. de Morisson Valeriano, J. C. Mendes, I. K. Jeck, W. H. dos Santos, A. L. de Oliveira, W. U. Mohriak, First petrologic data for vitória seamount, vitória-trindade ridge, south atlantic: A contribution to the trindade mantle plume evolution. *J. South Am. Earth Sci.* **109**, 103304 (2021).

97. K. Sreejith, K. Krishna, Magma production rate along the Ninetyeast Ridge and its relationship to Indian plate motion and Kerguelen hot spot activity. *Geophys. Res. Lett.* **42**, 1105–1112 (2015).

98. E. Bredow, B. Steinberger, Variable melt production rate of the Kerguelen hotspot due to long-term plume-ridge interaction. *Geophys. Res. Lett.* **45**, 126–136 (2018).

99. S. Homrighausen, K. Hoernle, J.-A. Wartho, F. Hauff, R. Werner, Do the 85°E Ridge and Conrad Rise form a hotspot track crossing the Indian Ocean? *Lithos* **398-399**, 106234 (2021).

100. A. A. P. Koppers, H. Staudigel, J. R. Wijbrans, M. S. Pringle, The Magellan seamount trail: Implications for Cretaceous hotspot volcanism and absolute Pacific plate motion. *Earth Planet. Sci. Lett.* **163**, 53–68 (1998).
101. A. A. P. Koppers, H. Staudigel, M. S. Pringle, J. R. Wijbrans, Short-lived and discontinuous intraplate volcanism in the South Pacific: Hot spots or extensional volcanism? *Geochem. Geophys. Geosyst.* **4** (2003).
102. V. Clouard, A. Bonneville, P.-Y. Gillot, The tarava seamounts: A newly characterized hotspot chain on the south pacific superswell. *Earth Planet. Sci. Lett.* **207**, 117–130 (2003).
103. R. A. Duncan, R. A. Keller, Radiometric ages for basement rocks from the Emperor Seamounts, ODP Leg 197. *Geochem. Geophys. Geosyst.* **5** (2004).
104. V. Clouard, A. Bonneville, Ages of seamounts, islands, and plateaus on the Pacific plate. *Special Pap. Geol. Soc. Am.* **388**, 71 (2005).
105. W. D. Sharp, D. A. Clague, 50-Ma initiation of Hawaiian-Emperor bend records major change in Pacific plate motion. *Science* **313**, 1281–1284 (2006).
106. A. A. P. Koppers, H. Staudigel, J. P. Morgan, R. A. Duncan, Nonlinear $^{40}Ar/^{39}Ar$ age systematics along the Gilbert Ridge and Tokelau Seamount Trail and the timing of the Hawaii-Emperor Bend. *Geochem. Geophys. Geosyst.* **8** (2007).
107. C. Adam, A. Bonneville, No thinning of the lithosphere beneath northern part of the Cook-Austral volcanic chains. *J. Geophys. Res. Solid Earth* **113** (2008).
108. J. Miyata, H. Takayanagi, A. Ishigaki, N. Hirano, S. Shiokawa, A. Nishimura, T. Nakazawa, T. Ishikawa, K. Nagaishi, H. Tokuyama, A. Ishiwatari, Y. Iryu, Tectonic implications of carbonate deposits on the eastern slope of the Hahajima Seamount in the collision zone between the Izu-Bonin Arc on the Philippine Sea Plate and the Ogasawara Plateau on the Pacific Plate. *Island Arc* **29**, e12368 (2020).
109. N. Hirano, H. Sumino, T. Morishita, S. Machida, T. Kawano, K. Yasukawa, T. Hirata, Y. Kato, T. Ishii , A Paleogene magmatic overprint on Cretaceous seamounts of the western Pacific. *Island Arc* **30**, e12386 (2021).
110. R. Pockalny, G. Barth, B. Eakins, K. A. Kelley, C. Wertman, Multiple melt source origin of the Line Islands (Pacific Ocean). *Geology* **49**, 1358–1362 (2021).
111. C. Cordier, H. Delavault, C. Chauvel, Geochemistry of the Society and Pitcairn-Gambier mantle plumes: What they share and do not share. *Geochim. Cosmochim. Acta* **306**, 362–384 (2021).

112. K. Hoernle, A. Schwindrofska, R. Werner, P. van den Bogaard, F. Hauff, G. Uenzelmann-Neben, D. Garbe-Schönberg, Tectonic dissection and displacement of parts of Shona hotspot volcano 3500 km along the Agulhas-Falkland Fracture Zone. *Geology* **44**, 263–266 (2016).
113. N. Parsiegla, K. Gohl, G. Uenzelmann-Neben, The Agulhas Plateau: Structure and evolution of a large igneous province. *Geophys. J. Int.* **174**, 336–350 (2008).
114. S. E. Bryan, L. Ferrari, Large igneous provinces and silicic large igneous provinces: Progress in our understanding over the last 25 years. *GSA Bull.* **125**, 1053–1078 (2013).
115. N. Youbi, R. E. Ernst, U. Söderlund, M. A. Boumehdi, A. A. Lahna, C. C. G. Tassinari, W. El Moume, M. K. Bensalah, The Central Iapetus magmatic province: An updated review and link with the ca. 580 Ma Gaskiers glaciation, in *Geological Society of America Special Paper 544: Mass Extinctions, Volcanism, and Impacts: New Developments*, T. Adatte, D. Bond, G. Kelle, eds. (Geological Society of America, 2020).
116. A. Vorontsov, V. Yarmolyuk, S. Dril, R. Ernst, O. Perfilova, O. Grinev, T. Komaritsyna, Magmatism of the Devonian Altai-Sayan Rift System: Geological and geochemical evidence for diverse plume-lithosphere interactions. *Gondw. Res.* **89**, 193–219 (2021).
117. A. J. Schaeffer, S. Lebedev, Imaging the North American continent using waveform inversion of global and USArray data. *Earth Planet. Sci. Lett.* **402**, 26–41 (2014).
118. N. L. Celli, S. Lebedev, A. J. Schaeffer, C. Gaina, African cratonic lithosphere carved by mantle plumes. *Nat. Commun.* **11**, 92 (2020).
119. E. Debayle, F. Dubuffet, S. Durand, An automatically updated S-wave model of the upper mantle and the depth extent of azimuthal anisotropy. *Geophys. Res. Lett.* **43**, 674–682 (2016).
120. K. Priestley, D. McKenzie, T. Ho, A lithosphere–asthenosphere boundary—A global model derived from multimode surface-wave tomography and petrology, in *Lithospheric Discontinuities*, H. Yuan, B. Romanowicz, eds. (John Wiley and Sons, 2018), chap. 6, pp. 111–124.
121. T. Ho, K. Priestley, E. Debayle, A global horizontal shear velocity model of the upper mantle from multimode love wave measurements. *Geophys. J. Int.* **207**, 542–561 (2016).
122. I. M. Artemieva, Global 1 × 1 thermal model tc1 for the continental lithosphere: Implications for lithosphere secular evolution. *Tectonophysics* **416**, 245–277 (2006).
123. X. Liu, N. Qiu, N. Søager, X. Fu, R. Liu, Geochemistry of late permian basalts from boreholes in the sichuan basin, sw China: Implications for an extension of the emeishan large igneous province. *Chem. Geol.* **588**, 120636 (2022).

124. H. Zhou, K. Hoernle, J. Geldmacher, F. Hauff, D. Garbe-Schönberg, S. Jung, I. Bindeman, Enriched mantle one (emi) type carbonatitic volcanism in namibia: Evidence for a concentrically-zoned Etendeka plume head. *Gondw. Res.* **109**, 239–252 (2022).
125. J. P. Pu, F. A. Macdonald, M. D. Schmitz, R. H. Rainbird, W. Bleeker, B. A. Peak, R. M. Flowers, P. F. Hoffman, M. Rioux, M. A. Hamilton, Emplacement of the Franklin large igneous province and initiation of the Sturtian Snowball Earth. *Sci. Adv.* **8**, eadc9430 (2022).
126. M. I. Kuzmin, V. V. Yarmolyuk, V. A. Kravchinsky, Phanerozoic hot spot traces and paleogeographic reconstructions of the siberian continent based on interaction with the African large low shear velocity province. *Earth Sci. Rev.* **102**, 29–59 (2010).
127. O. P. Polyansky, A. V. Prokopiev, O. V. Koroleva, M. D. Tomshin, V. V. Reverdatto, A. Y. Selyatitsky, A. V. Travin, D. A. Vasiliev, Temporal correlation between dyke swarms and crustal extension in the middle Palaeozoic Vilyui rift basin, Siberian platform. *Lithos* **282-283**, 45–64 (2017).
128. V. Puchkov, R. E. Ernst, M. A. Hamilton, U. Söderlund, N. Sergeeva, A Devonian >2000-km-long dolerite dyke swarm-belt and associated basalts along the Urals-Novozemelian fold-belt: Part of an East-European (Baltica) LIP tracing the Tuzo Superswell. *GFF* **138**, 6–16 (2016).
129. D. A. McConnell, M. J. Goydas, G. N. Smith, J. P. Chitwood, Morphology of the frontal fault zone, southwest oklahoma: Implications for deformation and deposition in the wichita uplift and anadarko basin. *Geology* **18**, 634 (1990).
130. L. Shumlyanskyy, A. Nosova, K. Billström, U. Söderlund, P. G. Andréasson, O. Kuzmenkova, The U–Pb zircon and baddeleyite ages of the Neoproterozoic Volyn Large Igneous Province: Implication for the age of the magmatism and the nature of a crustal contaminant. *GFF* **138**, 17–30 (2016).
131. R. S. White, Mantle plume origin for the karoo and ventersdorp flood basalts, south africa. *South Afr. J. Geol.* **100**, 271–282 (1997).
132. M. A. Coble, G. A. Mahood, Initial impingement of the yellowstone plume located by widespread silicic volcanism contemporaneous with columbia river flood basalts. *Geology* **40**, 655–658 (2012).
133. Y.-G. Xu, X. Wei, Z.-Y. Luo, H.-Q. Liu, J. Cao, The early permian tarim large igneous province: Main characteristics and a plume incubation model. *Lithos* **204**, 20–35 (2014).
