# Supplementary material for: Destruction and regrowth of lithospheric mantle beneath large igneous provinces
Source: Sci Adv. 2023 Sep 6;9(36):eadf6216. doi: 10.1126/sciadv.adf6216 (PMC11811892; doi:10.1126/sciadv.adf6216)
Supplement: Supplementary file 2 — Data files S1 to S6 [file sciadv.adf6216_data_files_s1_to_s6.zip › Data_S6.pdf]

-

Database References: *"Destruction and regrowth of lithospheric mantle beneath large igneous provinces"*

Simon N. Stephenson,*,[1], Patrick W. Ball,[2] & Fred D. Richards,[3]

1. Department of Earth Sciences, University of Oxford, Oxford, UK.
2. Department of Geosciences, Colorado State University, Fort Collins, Colorado, USA.
3. Department of Earth Science and Engineering, Imperial College London, London, UK

*simon.stephenson@earth.ox.ac.uk

Summary

Here we list references used to construct two databases and to carry out tests of our results. First we list references used to obtain the map of large igneous provinces. Secondly, we list the references for lithospheric thickness models that we use to test our lithospheric thickness - age relationship. Finally, we list the references used to construct the database of continental LIP eruption centres.

1 Large igneous province database

Below we list references used to adjust polygon outlines and ages for intraplate magmatic provinces. Please see Supplementary Information Section S3 and Figure S2 for further details.

1.1 Oceanic intraplate magmatic provinces

References for Oceanic provinces are listed by ocean.

1.1.1 Arctic

Døssing, A., Jackson, H. R., Matzka, J., Einarsson, I., Rasmussen, T. M., Olesen, A. V., & Brozena, J. (2013). On the origin of the Amerasia Basin and the High Arctic Large Igneous Province—results of new aeromagnetic data. *Earth and Planetary Science Letters*, *363*, 219–230.

Riefstahl, F., Estrada, S., Geissler, W. H., Jokat, W., Stein, R., Kämpf, H., Dulski, P., Naumann, R., & Spiegel, C. (2013). Provenance and characteristics of rocks from the Yermak Plateau, Arctic Ocean: Petrographic, geochemical and geochronological constraints. *Marine Geology*, *343*, 125–145.

Weber, J. (1990). The structures of the Alpha Ridge, Arctic Ocean and Iceland-Faeroe Ridge, North Atlantic: comparisons and implications for the evolution of the Canada Basin. *Marine Geology*, *93*, 43–68.

1.1.2 Atlantic

Duncan, R. A. (1984). Age progressive volcanism in the new england seamounts and the opening of the central atlantic ocean. *Journal of Geophysical Research: Solid Earth*, *89*(B12), 9980–9990.

Homrighausen, S., Hoernle, K., Hauff, F., Wartho, J.-A., van den Bogaard, P., & Garbe-Schönberg, D. (2019). New age and geochemical data from the Walvis Ridge: The temporal and spatial diversity of South Atlantic intraplate volcanism and its possible origin. *Geochimica et Cosmochimica Acta*, *245*, 16–34.

Maia, T. M., dos Santos, A. C., Rocha-Júnior, E. R. V., de Morisson Valeriano, C., Mendes, J. C., Jeck, I. K., dos Santos, W. H., de Oliveira, A. L., & Mohriak, W. U. (2021). First petrologic data for vitória seamount, vitória-trindade ridge, south atlantic: A contribution to the trindade mantle plume evolution. *Journal of South American Earth Sciences*, *109*, 103304.

Santos, R. V., Ganade, C. E., Lacasse, C. M., Costa, I. S., Pessanha, I., Frazão, E. P., Dantas, E. L., & Cavalcante, J. A. (2019). Dating Gondwanan continental crust at the Rio Grande Rise, South Atlantic. *Terra Nova*, *31*(5), 424–429.

1.1.3 Indian

Bredow, E., & Steinberger, B. (2018). Variable melt production rate of the Kerguelen hotspot due to long-term plume-ridge interaction. *Geophysical Research Letters*, *45*(1), 126–136.

Homrighausen, S., Hoernle, K., Wartho, J.-A., Hauff, F., & Werner, R. (2021). Do the 85°E Ridge and Conrad Rise form a hotspot track crossing the Indian Ocean? *Lithos*, *398*, 106234.

Sreejith, K., & Krishna, K. (2015). Magma production rate along the Ninetyeast Ridge and its relationship to Indian plate motion and Kerguelen hot spot activity. *Geophysical Research Letters*, *42*(4), 1105–1112.

1.1.4 Pacific

Adam, C., & Bonneville, A. (2008). No thinning of the lithosphere beneath northern part of the Cook-Austral volcanic chains. *Journal of Geophysical Research: Solid Earth*, *113*(B10).

Clouard, V., & Bonneville, A. (2005). Ages of seamounts, islands, and plateaus on the Pacific plate. *Special Papers-Geological Society of America*, *388*, 71.

Clouard, V., Bonneville, A., & Gillot, P.-Y. (2003). The tarava seamounts: A newly characterized hotspot chain on the south pacific superswell. *Earth and Planetary Science Letters*, *207*(1-4), 117–130.

Cordier, C., Delavault, H., & Chauvel, C. (2021). Geochemistry of the Society and Pitcairn-Gambier mantle plumes: What they share and do not share. *Geochimica et Cosmochimica Acta*, *306*, 362–384.

Duncan, R. A., & Keller, R. A. (2004). Radiometric ages for basement rocks from the Emperor Seamounts, ODP Leg 197. *Geochemistry, Geophysics, Geosystems*, *5*(8).

Hirano, N., Sumino, H., Morishita, T., Machida, S., Kawano, T., Yasukawa, K., Hirata, T., Kato, Y., & Ishii, T. (2021). A Paleogene magmatic overprint on Cretaceous seamounts of the western Pacific. *Island Arc*, *30*(1), e12386.

Huppert, K. L., Perron, J. T., & Royden, L. H. (2020). Hotspot swells and the lifespan of volcanic ocean islands. *Science advances*, *6*(1), eaaw6906.

Koppers, A. A., Staudigel, H., Phipps Morgan, J., & Duncan, R. A. (2007). Nonlinear ^{40}Ar/^{39}Ar age systematics along the Gilbert Ridge and Tokelau Seamount Trail and the timing of the Hawaii-Emperor Bend. *Geochemistry, Geophysics, Geosystems*, *8*(6).

Koppers, A. A., Staudigel, H., Pringle, M. S., & Wijbrans, J. R. (2003). Short-lived and discontinuous intraplate volcanism in the South Pacific: Hot spots or extensional volcanism? *Geochemistry, Geophysics, Geosystems*, *4*(10).

Koppers, A. A., Staudigel, H., Wijbrans, J. R., & Pringle, M. S. (1998). The Magellan seamount trail: implications for Cretaceous hotspot volcanism and absolute Pacific plate motion. *Earth and Planetary Science Letters*, *163*(1-4), 53–68.

Miyata, J., Takayanagi, H., Ishigaki, A., Hirano, N., Shiokawa, S., Nishimura, A., Nakazawa, T., Ishikawa, T., Nagaishi, K., Tokuyama, H., Ishiwatari, A., & Iryu, Y. (2020). Tectonic implications of carbonate deposits on the eastern slope of the Hahajima Seamount in the collision zone between the Izu–Bonin Arc on the Philippine Sea Plate and the Ogasawara Plateau on the Pacific Plate. *Island Arc*, *29*(1).

Pockalny, R., Barth, G., Eakins, B., Kelley, K. A., & Wertman, C. (2021). Multiple melt source origin of the Line Islands (Pacific Ocean). *Geology*, *49*(11), 1358–1362.

Sharp, W. D., & Clague, D. A. (2006). 50-Ma initiation of Hawaiian-Emperor bend records major change in Pacific plate motion. *Science*, *313*(5791), 1281–1284.

1.1.5 Southern

Hoernle, K., Schwindrofska, A., Werner, R., van den Bogaard, P., Hauff, F., Uenzelmann-Neben, G., & Garbe-Schönberg, D. (2016). Tectonic dissection and displacement of parts of Shona hotspot volcano 3500 km along the Agulhas-Falkland Fracture Zone. *Geology*, *44*(4), 263–266.

Parsiegla, N., Gohl, K., & Uenzelmann-Neben, G. (2008). The Agulhas Plateau: Structure and evolution of a large igneous province. *Geophysical Journal International*, *174*(1), 336–350.

1.2 Continental large Igneous Provinces

Bryan, S. E., & Ferrari, L. (2013). Large igneous provinces and silicic large igneous provinces: Progress in our understanding over the last 25 years. *GSA Bulletin*, *125*(7-8), 1053–1078.

Coffin, M. F., Duncan, R. A., Eldholm, O., Fitton, J. G., Frey, F. A., Larsen, H. C., Mahoney, J. J., Saunders, A. D., Schlich, R., & Wallace, P. J. (2006). Large igneous provinces and scientific ocean drilling: Status quo and a look ahead. *Oceanography*, *19*(4), 150–160.

Coffin, M. F., & Eldholm, O. (1994). Large igneous provinces: Crustal structure, dimensions, and external consequences. *Reviews of Geophysics*, *32*(1), 1–36.

Ernst, R. E., Bond, D. P., Zhang, S.-H., Buchan, K. L., Grasby, S. E., Youbi, N., El Bilali, H., Bekker, A., & Doucet, L. S. (2021). Large igneous province record through time and implications for secular environmental changes and geological time-scale boundaries. In R. E. Ernst, A. J. Dickson, & A. Becker (Eds.), *Large igneous provinces: A driver of global environmental and biotic changes* (pp. 3–26). American Geophysical Union (AGU).

Vorontsov, A., Yarmolyuk, V., Dril, S., Ernst, R., Perfilova, O., Grinev, O., & Komaritsyna, T. (2021). Magmatism of the devonian altai-sayan rift system: Geological and geochemical evidence for diverse plume-lithosphere interactions. *Gondwana Research*, *89*, 193–219.

Youbi, N., Ernst, R. E., Söderlund, U., Boumehdi, M. A., Lahna, A. A., Tassinari, C. C. G., El Moume, W., & Bensalah, M. K. (2020). The Central Iapetus magmatic province: An updated review and link with the ca. 580 Ma Gaskiers glaciation. In T. Adatte, D. Bond, & G. Kelle (Eds.), *Geological Society of America Special Paper 544: Mass Extinctions, Volcanism, and Impacts: New Developments*. Geological Society of America.

2 Alternative lithospheric thickness models

2.0.1 SLNAAFSA

Model based upon blending a global V_S tomographic model with high-resolution regional models for Africa, South Atlantic and North America.

Celli, N. L., Lebedev, S., Schaeffer, A. J., & Gaina, C. (2020). African cratonic lithosphere carved by mantle plumes. *Nature communications*, *11*(1), 1–10.

Celli, N. L., Lebedev, S., Schaeffer, A. J., Ravenna, M., & Gaina, C. (2020). The upper mantle beneath the South Atlantic Ocean, South America and Africa from waveform tomography with massive data sets. *Geophysical Journal International*, *221*(1), 178–204.

Hoggard, M. J., Czarnota, K., Richards, F. D., Huston, D. L., Jaques, A. L., & Ghelichkhan, S. (2020). Global distribution of sediment-hosted metals controlled by craton edge stability. *Nature Geoscience*, *13*(7), 504–510.

Schaeffer, A. J., & Lebedev, S. (2013). Global shear speed structure of the upper mantle and transition zone. *Geophysical Journal International*, *194*(1), 417–449.

Schaeffer, A., & Lebedev, S. (2014). Imaging the north american continent using waveform inversion of global and usarray data. *Earth and Planetary Science Letters*, *402*, 26–41.

2.1 3D2015-07Sv

Debayle, E., Dubuffet, F., & Durand, S. (2016). An automatically updated s-wave model of the upper mantle and the depth extent of azimuthal anisotropy. *Geophysical Research Letters*, *43*(2), 674–682.

Hoggard, M. J., Czarnota, K., Richards, F. D., Huston, D. L., Jaques, A. L., & Ghelichkhan, S. (2020). Global distribution of sediment-hosted metals controlled by craton edge stability. *Nature Geoscience*, *13*(7), 504–510.

2.2 CAM2016

Ho, T., Priestley, K., & Debayle, E. (2016). A global horizontal shear velocity model of the upper mantle from multimode love wave measurements. *Geophysical journal international*, *207*(1), 542–561.

Hoggard, M. J., Czarnota, K., Richards, F. D., Huston, D. L., Jaques, A. L., & Ghelichkhan, S. (2020). Global distribution of sediment-hosted metals controlled by craton edge stability. *Nature Geoscience*, *13*(7), 504–510.

Priestley, K., McKenzie, D., & Ho, T. (2019). A lithosphere-asthenosphere boundary-a global model derived from multimode surface-wave tomography and petrology. In H. Yuan & B. Romanowicz (Eds.), *Lithospheric discontinuities* (pp. 111–124). John Wiley; Sons.

2.3 A2006

Artemieva, I. M. (2006). Global 1× 1 thermal model tc1 for the continental lithosphere: Implications for lithosphere secular evolution. *Tectonophysics*, *416*(1-4), 245–277.

3 Large igneous province eruption centres

Buchan, K. L., & Ernst, R. E. (2018). A giant circumferential dyke swarm associated with the high arctic large igneous province (halip). *Gondwana Research*, *58*, 39–57.

Coble, M. A., & Mahood, G. A. (2012). Initial impingement of the yellowstone plume located by widespread silicic volcanism contemporaneous with columbia river flood basalts. *Geology*, *40*(7), 655–658.

Kuzmin, M. I., Yarmolyuk, V. V., & Kravchinsky, V. A. (2010). Phanerozoic hot spot traces and paleogeographic reconstructions of the siberian continent based on interaction with the african large low shear velocity province. *Earth-Science Reviews*, *102*(1-2), 29–59.

Liu, X., Qiu, N., Søager, N., Fu, X., & Liu, R. (2022). Geochemistry of late permian basalts from boreholes in the sichuan basin, sw china: Implications for an extension of the emeishan large igneous province. *Chemical Geology*, *588*, 120636.

McConnell, D. A., Goydas, M. J., Smith, G. N., & Chitwood, J. P. (1990). Morphology of the frontal fault zone, southwest oklahoma: Implications for deformation and deposition in the wichita uplift and anadarko basin. *Geology*, *18*(7), 634–637.

Polyansky, O. P., Prokopiev, A. V., Koroleva, O. V., Tomshin, M. D., Reverdatto, V. V., Selyatitsky, A. Y., Travin, A. V., & Vasiliev, D. A. (2017). Temporal correlation between dyke swarms and crustal extension in the middle palaeozoic vilyui rift basin, siberian platform. *Lithos*, *282*, 45–64.

Pu, J. P., Macdonald, F. A., Schmitz, M. D., Rainbird, R. H., Bleeker, W., Peak, B. A., Flowers, R. M., Hoffman, P. F., Rioux, M., & Hamilton, M. A. (2022). Emplacement of the franklin large igneous province and initiation of the sturtian snowball earth. *Science Advances*, *8*(47), eadc9430.

Puchkov, V., Ernst, R. E., Hamilton, M. A., Söderlund, U., & Sergeeva, N. (2016). A devonian¿ 2000-km-long dolerite dyke swarm-belt and associated basalts along the urals-novozemelian fold-belt: Part of an east-european (baltica) lip tracing the tuzo superswell. *GFF*, *138*(1), 6–16.

Saunders, A., Jones, S., Morgan, L., Pierce, K., Widdowson, M., & Xu, Y. (2007). Regional uplift associated with continental large igneous provinces: The roles of mantle plumes and the lithosphere. *Chemical Geology*, *241*(3-4), 282–318.

Sembroni, A., Faccenna, C., Becker, T. W., Molin, P., & Abebe, B. (2016). Long-term, deep-mantle support of the ethiopia-yemen plateau. *Tectonics*, *35*(2), 469–488.

Shumlyanskyy, L., Nosova, A., Billström, K., Söderlund, U., Andréasson, P.-G., & Kuzmenkova, O. (2016). The u–pb zircon and baddeleyite ages of the neoproterozoic volyn large igneous province: Implication for the age of the magmatism and the nature of a crustal contaminant. *Gff*, *138*(1), 17–30.

White, R. (1997). Mantle plume origin for the karoo and ventersdorp flood basalts, south africa. *South African Journal of Geology*, *100*(4), 271–282.

Xu, Y.-G., Wei, X., Luo, Z.-Y., Liu, H.-Q., & Cao, J. (2014). The early permian tarim large igneous province: Main characteristics and a plume incubation model. *Lithos*, *204*, 20–35.

Zhou, H., Hoernle, K., Geldmacher, J., Hauff, F., Garbe-Schönberg, D., Jung, S., & Bindeman, I. (2022). Enriched mantle one (emi) type carbonatitic volcanism in namibia: Evidence for a concentrically-zoned etendeka plume head. *Gondwana Research*, *109*, 239–252.
